# Tailored D‐π Conjugation Boosts Piezocatalytic CO_2_ Reduction in a Platinum(II)‐Acetylide Framework

**DOI:** 10.1002/advs.75849

**Published:** 2026-05-28

**Authors:** Mude Zhu, Yingtang Zhou, Kai Wang, Xiaoyun Fan, Yang Ding, Wai‐Yeung Wong, Linli Xu

**Affiliations:** ^1^ Department of Applied Biology and Chemical Technology and Research Institute for Smart Energy The Hong Kong Polytechnic University Kowloon Hong Kong SAR P. R. China; ^2^ Marine Science and Technology College Zhejiang Ocean University Zhoushan P. R. China; ^3^ School of Physics Science and Information Technology Liaocheng University Liaocheng P. R. China; ^4^ College of Environment and Climate Guangdong Provincial Key Laboratory of Environmental Pollution and Health Jinan University Guangzhou P. R. China; ^5^ Engineering Research Center for Semiconductor Integrated Technology Institute of Semiconductors Chinese Academy of Sciences Beijing P. R. China

**Keywords:** CO_2_ reduction, electron delocalization, mechanical energy conversion, metal‐acetylide frameworks, two‐dimensional piezocatalysts

## Abstract

Piezocatalytic CO_2_ reduction offers a sustainable route for converting mechanical energy into chemical fuels. However, its practical implementation demands catalysts that concurrently exhibit a strong piezoelectric response and high catalytic activity. Herein, we report a one‐pot synthesis of well‐defined metal–acetylide frameworks (TTED‐M‐AFs; M = Pt, Pd, Ni) incorporating M^II^(PEt_3_)_2_ units into an extended graphdiyne‐type scaffold via robust ─C≡C─M^II^(PEt_3_)_2_─C≡C─ linkages. These molecular metal‐bis(acetylide) motifs function as intrinsic active sites for CO_2_ reduction. Under mechanical agitation, the Pt^II^‐based framework achieves a CO production rate of 72.03 µmol g^−1^ h^−1^ with 92.4% selectivity, outperforming its Pd^II^ and Ni^II^ analogues by factors of 1.17 and 1.73, respectively—a trend consistent with their piezoelectric coefficients (*d*_33_ = 35, 21.9, and 12.5 pm V^−1^). Combined experimental and theoretical analyses reveal that the piezoelectric field in TTED‐Pt‐AF enhances CO_2_ adsorption and promotes local electron accumulation, thereby lowering the activation energy barrier. Furthermore, in situ high‐pressure FT‐IR spectroscopy demonstrates that the Pt^II^‐bis(acetylide) centers exhibit superior electronic synergy with the tetraphenylene‐derived π‐conjugated matrix under mechanical stress, inducing pronounced d–π orbital hybridization. The exceptional piezocatalytic performance, coupled with a scalable synthesis, underscores the promise of metal–acetylide frameworks as efficient platforms for mechano‐driven CO_2_ valorization.

## Graphical abstract

The highly conjugated metal^II^‐acetylide framework demonstrates efficient ultrasound‐driven conversion of CO_2_ to CO. The piezoelectric field generated by ultrasound facilitates electron transfer from the tetraphenylene π‐column to the M^II^‐bis(acetylide) moiety, enabling CO_2_ activation. Compared to Pd^II^‐ and Ni^II^‐based analogues, the stronger d–π orbital hybridization within Pt^II^‐bis(acetylide) creates a more extensively delocalized and highly polarizable electronic structure, thereby accelerating electron migration and enhancing catalytic performance.

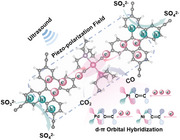

## 1 Introduction

**FIGURE 1:**
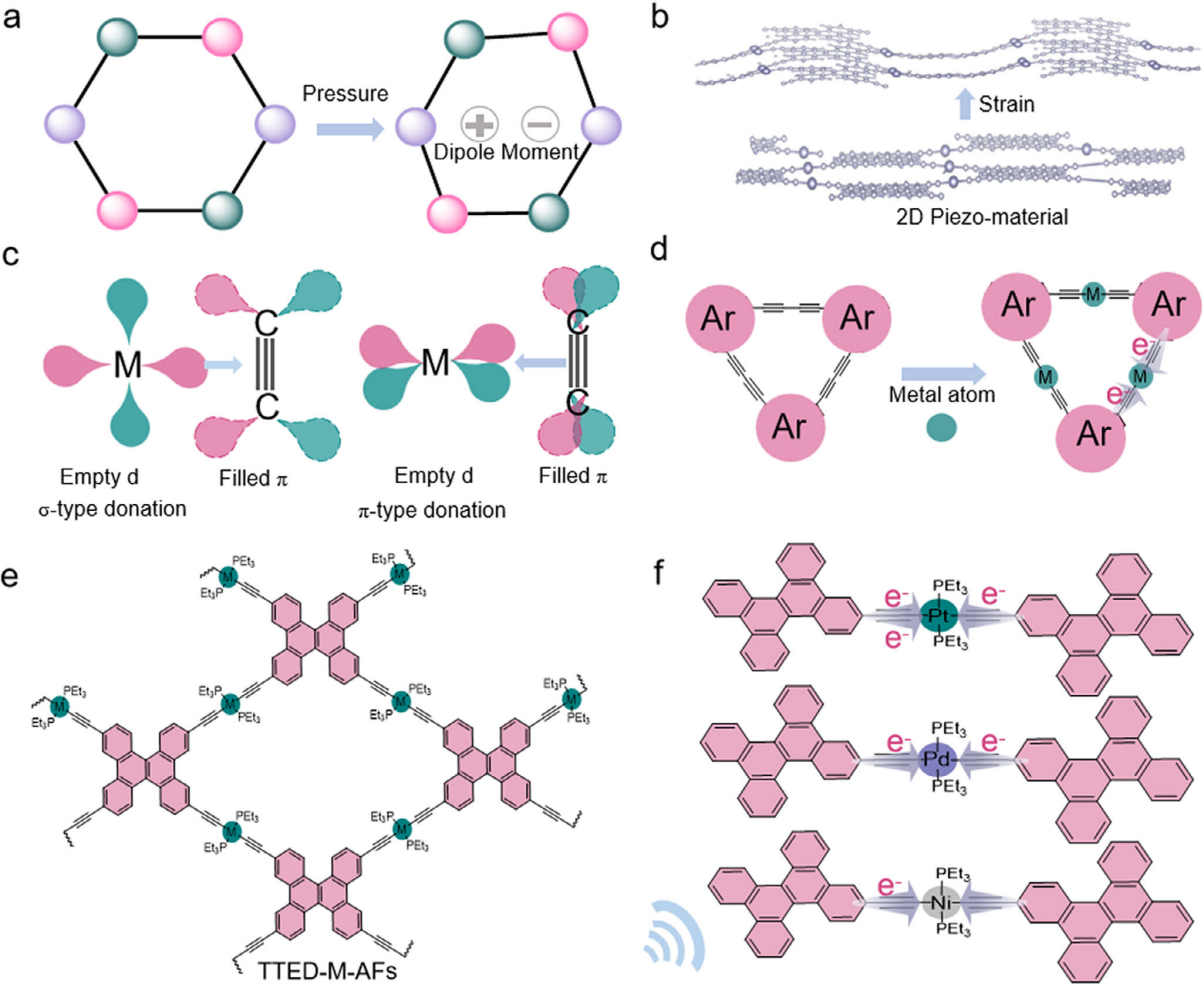
(a) Schematic illustration of dipole moment generation induced by applied pressure. (b) Strain‐induced deformation behavior of 2D piezoelectric materials. (c) Metal‐acetylene bonding interactions highlighting orbital overlap and coordination. (d) Metal‐centered electronic redistribution within metal‐acetylide frameworks. (e) Molecular structure of TTED‐M‐AFs (M = Pt, Pd, Ni) depicting key framework motifs. (f) Comparative electron transfer efficiencies across TTED‐Pt‐AF, TTED‐Pd‐AF, and TTED‐Ni‐AF under applied mechanical pressure.

The catalytic reduction of CO_2_ into value‐added chemicals represents a critical challenge in addressing climate change and advancing sustainable energy technologies. The development of renewable energy systems is essential to mitigate CO_2_ emissions and enable efficient carbon capture and utilization [1]. Recently, piezocatalysis, which harnesses mechanical energy to drive CO_2_ reduction, has attracted considerable interest due to its ability to lower activation energy barriers and enhance reaction kinetics [2, 3, 4]. The piezoelectric effect, arising from materials lacking inversion symmetry, induces electric dipoles under mechanical deformation. This generates a polarization field that facilitates charge carrier separation and directional migration to catalytic active sites, thereby facilitating redox reactions (Figure 1a) [5].

The performance of the piezocatalytic CO_2_ reduction reaction (PCRR) is critically governed by the intrinsic piezoelectric properties of the catalyst, which regulate charge carrier generation, separation, transport, and recombination. To improve piezocatalytic efficiency, strategies such as ion doping, atomic deposition, and defect engineering have been employed to induce lattice distortions and enhance piezoelectric polarization [6, 7]. Two‐dimensional (2D) piezoelectric materials are particularly promising PCRR catalysts due to their high strain sensitivity and excellent charge carrier mobility (Figure 1b) [8]. For instance, 2D BiFeO_3_ nanosheets achieve a CO production rate of 28.7 µmol g^−^^1^ h^−^^1^, outperforming LaNiO_3_ nanoparticles and LaFeO_3_ nanospheres, owing to their superior flexibility and polarization under vibration.^2^ Despite these advances, the CO_2_ reduction efficiency of current 2D piezoelectric materials remains limited, and research in this area is still nascent [9, 10].

Graphdiynes (GDYs), a class of 2D carbon‐rich frameworks featuring abundant carbon‐carbon triple bonds (−C≡C−), exhibit distinctive structural and electronic properties advantageous for catalysis [11]. These linear, *sp*‑hybridized carbon chains provide an ideal platform for achieving strong d–π conjugation with transition‑metal centers and can be structurally modulated to introduce piezo‑responsive behavior through symmetry‑breaking strategies, such as via substitutional doping with S or NH_2_ groups [12, 13]. The rotational degree of freedom inherent to these acetylenic units further optimizes orbital overlap with metal states, a key design principle that informs the construction of metal‑coordinated frameworks rather than implying topological equivalence (Figure 1c) [14, 15]. These interactions enhance electronic conjugation and stabilize metal‐organic frameworks (MOFs), making them effective platforms for electron transfer [16]. A variety of metal‐coordinated GDY‐based frameworks, incorporating Ni [17], Cu [18], Fe [19], Pt [20], Pd [21], and Ag [22] centers via d–π electronic coupling have been synthesized, in which the metal atoms serve dual roles as electron mediators and catalytic active sites.

Building on these advances, our group has successful integrated transition metal (TM) moieties, including Hg, Ni(PR_3_)_2_, Pd(PR_3_)_2_, and Pt(PR_3_)_2_ (R = alkyl), into GDY frameworks via d–π orbital overlap, forming highly conjugated metal(II)‐acetylide frameworks (MAFs) for photocatalytic CO_2_ reduction [23, 24, 25]. These TM centers enhance CO_2_ adsorption and activation, improve reaction kinetics and selectivity, and promote favorable electronic redistribution to facilitate electron transfer. Moreover, TM incorporation can strengthen piezoelectric polarization by modulating electronic structure, atomic size effects, interfacial interactions, and lattice distortions [26, 27]. For example, TM‐doped MOFs such as Mg‐, Mn‐, and Ni‐modified Co‐MOFs exhibit improved dipole alignment and polarization [28], while Cu incorporation into NH_2_‐MIL‐125 raises the piezoelectric coefficient (*d_33_*) from 11.75 to 26.21 pm V^−1^ [29]. These results emphasize the pivotal role of TM moieties in optimizing electromechanical responses and catalytic efficacy. In our previous work, we reported a Ni^II^‐acetylide framework (HETP‐Ni‐AF) in which Ni(PEt_3_)_2_ units simultaneously boost polarization and PCRR activity (Figure 1d) [30]. Despite these advances, the influence of different TM units on the piezocatalytic performance of MAFs remains largely unexplored.

Herein, we synthesized a series of metal^II^‐acetylide frameworks, TTED‐Pt‐AF, TTED‐Pd‐AF, and TTED‐Ni‐AF, by incorporating Pt^II^(PEt_3_)_2_, Pd^II^(PEt_3_)_2_, and Ni^II^(PEt_3_)_2_ units, respectively, into a highly conjugated acetylide‐based GDY scaffold derived from 2, 7, 10, 15‐tetrakis(ethynyl)dibenzog, pchrysene (TTED) (Figure 1e). The dibenzog, pchrysene backbone was selected for its rigid structure and extended π‐conjugation, which facilitates efficient charge transport [31, 32]. Among the synthesized frameworks, TTED‐Pt‐AF exhibits superior PCRR activity and selectivity. The incorporation of Pt^II^(PEt_3_)_2_ units induces a stronger piezoelectric polarization field compared to Pd^II^‐ and Ni^II^‐analogues (Figure 1f). The energy levels of Pt 5*d* orbitals align more favorably with the π orbitals of the ligand than those of 4*d* (Pd) and 3*d* (Ni) analogues, enhancing orbital interactions and enabling efficient electron transfer [33]. The ─C≡C─Pt(PEt_3_)_2_─C≡C─ moieties serve as the primary active site for CO_2_ adsorption and catalysis, stabilizing key reactive intermediates while improving conversion efficiency and product selectivity. They provide a robust platform for mechanistic studies and offer a sustainable strategy for CO_2_ valorization via integrated piezoelectric and catalytic functionalities. Additionally, the key outcome is the establishment of a clear and predictable structure−property−performance relationship, namely, that d‐π overlap, piezoelectric response, and catalytic efficiency increase in the order Ni (3d) < Pd (4d) < Pt (5d), which transcends the superior performance of any single material. This mechanistic insight offers a foundational blueprint for designing high‐performance piezocatalysts, advancing both mechano‐energy conversion and sustainable carbon utilization technologies.

## 2 Results and Discussion

### 2.1 Synthesis and Structural Characterization of Metal‐Acetylide Frameworks

A series of 2D metal‐acetylide frameworks, denoted as TTED‐M‐AFs (M = Pt, Pd, Ni), were synthesized via a base‐catalyzed dehydrohalogenation reaction between the organic ligand 2,7,10,15‐tetrakis(ethynyl)dibenzo[g,p]chrysene (TTED) and the metal complex *trans*‐M(PEt_3_)_2_Cl_2_ (Schemes S1 and S2 and Figures S1 and S2). A metal‐free control framework, TTED‐GDY, was prepared under analogous conditions for comparison studies (Scheme S3).

**FIGURE 2:**
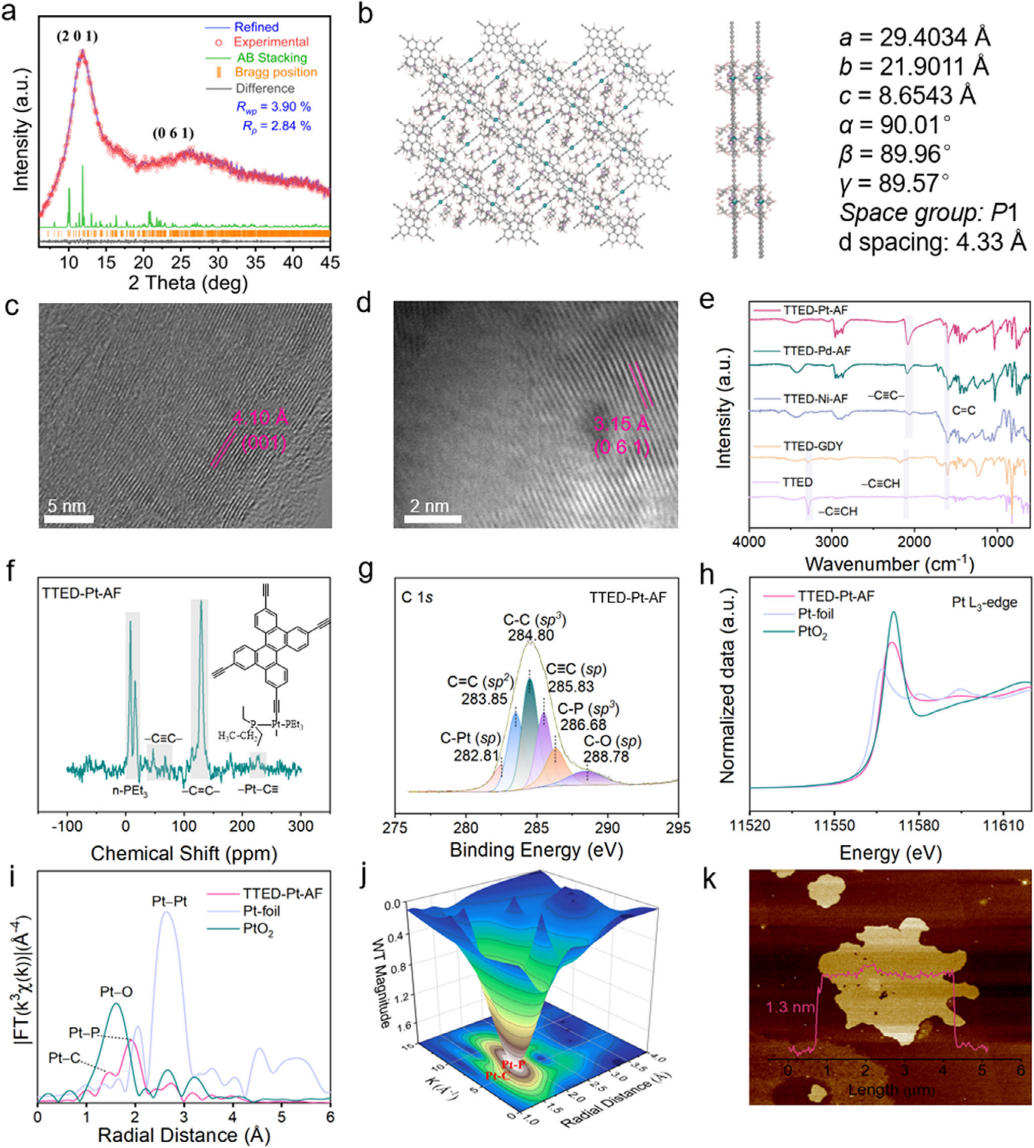
(a) Experimental PXRD pattern of TTED‐Pt‐AF (red), profile calculated by Pawley refinement (blue), and the residual difference (yellow), compared with the simulated pattern from the optimized structural model (green). Reflection positions are indicated by tick marks. (b) Approximate unit cell parameters from Pawley refinement of TTED‑Pt‑AF. (c) HR‐TEM image showing the interlayer spacing of TTED‐Pt‐AF. (d) HR‐TEM image corresponding to the (0 6 1) crystallographic plane of TTED‐Pt‐AF. (e) FTIR spectra of TTED‐M‐AFs, TTED‐GDY, and the pristine TTED ligand. (f) Solid‐state ^13^C CP‐MAS NMR spectrum of TTED‐Pt‐AF. (g) High‐resolution C 1s XPS spectrum of TTED‐Pt‐AF. (h) XANES spectra of TTED‐Pt‐AF at the Pt *L_3_*‐edge, with Pt foil and PtO_2_ as reference standards. (i) Fourier transform of Pt *L_3_*‐edge EXAFS for TTED‐Pt‐AF. (j) 3D wavelet transform of Pt *L_3_*‐edge EXAFS for TTED‐Pt‐AF using *k^3^*‐weighting. (k) AFM image depicting the morphology and thickness of TTED‐Pt‐AF nanosheets.

The local structural order of the frameworks was examined by powder x‑ray diffraction (PXRD). All TTED‑M‑AFs exhibit broad peaks indicative of low to moderate crystallinity, consistent with the formation of layered materials with significant stacking disorder. Pawley refinement was attempted assuming an AB stacking model (Figures S3, S4a and S5a). TTED‐Pt‐AF showed intense reflections at 2θ = 11.85° and 26.50°, corresponding to the (2 0 1) and (0 6 1) planes, respectively (Figure 2a). Refinement yielded unit cell parameters of *a* = 29.40 Å, *b* = 21.90 Å, *c* = 8.65 Å, and *α* = 90.01°, *β* = 89.96°, and *γ* = 89.57° (*R_wp_*  = 3.90%, *R_p_*  = 2.84%) (Figure 2b). For TTED‐Pd‐AF, reflections at 11.41° and 21.17° were assigned to the (2 2 0) and (1 0 2) planes, with unit cell dimensions *a* = 29.69 Å, *b* = 20.98 Å, *c* = 8.47 Å, and *α* = 90.02°, *β* = 89.97°, *γ* = 89.75° (*R_wp_*  = 4.61%, *R_p_*  = 5.24%) (Figure S4b,c). Similarly, TTED‐Ni‐AF displayed peaks at 10.28° and 20.96°, corresponding to the (2 2 0) and (0 0 2) planes, with unit cell parameters *a* = 29.69 Å, *b* = 20.97 Å, *c* = 8.39 Å, and *α* = 90.10°, *β* = 89.89, *γ* = 89.83° (*R_p_* = 3.40%, *R_wp_* = 3.66%) (Figure S5b,c). Obviously, the PXRD patterns of TTED‑Pt‑AF, TTED‑Pd‑AF, and TTED‑Ni‑AF are not identical; the relative intensity of the peak near 2θ = 12° varies significantly, and some peaks present in one pattern are absent or broadened in another. These differences likely arise from variations in coordination kinetics, defect density, and stacking disorder during synthesis. A systematic decrease in the interlayer spacing was observed from Pt (4.32 Å) to Pd (4.23 Å) to Ni (4.19 Å), consistent with the decreasing ionic radii of the metal centers. High‐resolution TEM (HR‑TEM) analysis of the internal flake regions shows faint fringe‑like features with measured spacings of 4.10, 4.08, and 3.93 Å for TTED‑Pt‑AF, TTED‑Pd‑AF, and TTED‑Ni‑AF, respectively. These spacings are consistent with the expected interlayer distances of layered metal‑acetylide frameworks. The measured values are presented as approximate local layer spacings due to their low crystallinity. Additional spacings of approximately 3.15 Å, 3.50 Å, and 3.55 Å were observed in HR‑TEM regions. They are broadly consistent with the local packing expected for a layered metal‑acetylide framework (Figures 2c,d and Figures S6 and S7). HR‐TEM of TTED‐Pt‐AF (Figure S8) shows occasional regions with weak contrast that could be consistent with local pore‐like features. To date, the successful synthesis of MAFs has been limited to a small group of transition metals (Hg^II^, Ni^II^, Pd^II^, and Pt^II^). Expanding this family to include other 3d, 4d, and 5d metals, therefore, represents a crucial direction for future research.

The formation of the ─C≡C─M(PEt_3_)_2_─C≡C─ coordination motif was verified by multiple spectroscopic techniques. Fourier transform infrared (FT ‐ IR) spectroscopy showed the disappearance of the terminal ─C≡CH stretching vibrations (observed at 2048 and 3282 cm^−1^ in the TTED monomer) and the emergence of new bands ─C≡C−M(PEt_3_)_2_─C≡C─ vibrations at 2081, 2094, and 2059 cm^−1^ for TTED‐Pt‐AF, TTED‐Pd‐AF, and TTED‐Ni‐AF, respectively [34, 35]. Additional signals corresponding to the ─C≡C─ (∼2080 cm^−1^) and ─CH_2_CH_3_ groups (2780−2980 cm^−1^) were also observed (Figure 2e). A consistent peak at ∼1647 cm^−1^ was assigned to conjugated C═C stretching vibrations [36]. Raman spectroscopy further confirmed the metal‐alkynyl bonding, as evidenced by ─C≡C─ stretching modes in the range of ∼2112–2208 cm^−1^ (Figure S9). Solid‐state ^13^C cross‐polarization magic‐angle spinning (CP‐MAS) nuclear magnetic resonance (NMR) spectra exhibited resonances for alkynyl carbons at 83.75 ppm and aromatic carbons of the dibenzochrysene unit at 137.67 ppm, along with a distinctive downfield signal at ∼236 ppm attributed to the −Pt−C≡ moiety (Figure 2f and Figure S10) [37, 38]. The coordination of M^II^(PEt_3_)_2_ units within the frameworks was further corroborated by solid‐state ^31^P NMR spectroscopy (Figure S11).

N_2_ adsorption‐desorption isotherms displayed type II behavior, with Brunauer–Emmett–Teller (BET) specific surface areas of 15.79, 124.82, and 17.44 m^2^ g^−1^ for TTED‐Pt‐AF, TTED‐Pd‐AF, and TTED‐Ni‐AF, respectively (Figure S12). The markedly higher specific surface area of TTED‐Pd‐AF is attributed to the optimal coordination kinetics of Pd^II^. This intermediate kinetic facilitates a slow, reversible assembly process, yielding highly crystalline frameworks with minimal defects and maximal porosity. In contrast, the faster kinetics of Ni^II^ promote the formation of defect‐rich structures, while the stronger Pt^II^–ligand bonds reduce assembly reversibility, potentially leading to interpenetration or pore collapse. This is corroborated by the lower bulk density and expanded powder volume of TTED‐Pd‐AF (Figure S13). The relatively low surface areas are attributed to pore blocking by the bulky PEt_3_ auxiliary ligands and possible framework aggregation, which limit pore accessibility. Pore size distributions centered around 0.78 nm aligned well with pore volumes of AB stacking mode calculated from density functional theory (DFT) (Figures S14–S16).

X‐ray photoelectron spectroscopy (XPS) analysis confirmed the surface elemental compositions, which were consistent with the proposed framework formulations, identifying the presence of metal, phosphorus, and carbon species. The high‐resolution C 1s spectrum of TTED‐Pt‐AF displayed deconvoluted peaks at binding energies of 282.81, 283.85, 284.80, 285.83, 286.68, and 288.78 eV, corresponding to C–Pt (*sp*), C–C (*sp* ^2^, *sp* ^3^, *sp*), C–P, and C–O bonding environments, respectively (Figure 2g) [23, 39]. Analogous metal–carbon bonding signatures were observed in TTED‐Ni‐AF, TTED‐Pd‐AF, and TTED‐GDY (Figures S17 and S18), corroborating successful metal incorporation and framework stability [40]. Additionally, XPS analysis confirms that the Ni, Pd, and Pt centers in TTED‐M‐AFs adopt the +2 oxidation state. The P 2p binding energies follow the trend TTED‐Ni‐AF > TTED‐Pd‐AF > TTED‐Pt‐AF, reflecting increasing metal‐to‐ligand π‐backdonation from the filled d‐orbitals of the metal center to the antibonding orbitals of the phosphine ligands. This backdonation enhances electron density on phosphorus, providing greater core‐electron shielding and thus lowering the P 2p binding energy. The effect is most pronounced for Pt owing to optimal overlap between its diffuse 5d orbitals and ligand acceptor orbitals, followed by Pd 4d and Ni (contracted 3d), which in turn stabilize charge‐transfer states and boost catalytic performance.

Pt *L*_3_‐edge x‐ray absorption spectroscopy (XAS) elucidates the local electronic structure and coordination environment. The XANES spectrum of TTED‐Pt‐AF displays an absorption edge intermediate between Pt foil and PtO_2_, consistent with an average Pt^II^ oxidation state (Figure 2h). High‐resolution XPS confirms Pt^II^ (Pt 4f_7/2_ = 72.56 eV), corroborated by synchrotron Pt *L*_3_‐edge XANES, where the white‐line position and edge energy match Pt^II^ standards and differ markedly from Pt(0) and Pt^IV^ references (Figures 2h,i and Figure S17). The fitting results yield an average oxidation state of approximately +1.85, closely aligning with the expected value for a Pt^II^ center and indicating a slightly reduced state (Figure S19). Fourier‐transform EXAFS reveals Pt–C (∼1.5 Å) and Pt–P (∼1.9 Å) coordination shells, without Pt–Pt (∼2.6 Å) or Pt–O (∼1.6 Å) contributions, confirming atomic Pt dispersion (Figures 2i and Figure S20a–c). Quantitative fits to the *k* ^3^‐weighted *χ* ^(k)^‐function (Figure S20d–g and Table S1) and wavelet transform analysis (single maximum at ∼5.55 Å^−1^ ascribed to Pt–C/P; Figure 2j and Figure S20h–i) corroborate this structure, attributable to strong *sp*‐C–Pt 5d orbital overlap that elevates Pt migration barriers and stabilizes isolated sites.

Atomic force microscopy (AFM), scanning electron microscopy (SEM), transmission electron microscopy (TEM), and energy‐dispersive X‐ray spectroscopy (EDS) confirm layered nanosheet morphologies with uniform elemental distribution in all TTED‐M‐AFs and TTED‐GDY (Figures S21–S25). The TTED ligand features a large, planar, and rigidly π‐conjugated aromatic backbone, which inherently favors in‐plane propagation when linked via linear ditopic connectors. This geometry strongly promotes the formation of stable, sheet‐like 2D frameworks. The extended conjugation not only facilitates charge delocalization within the layers but also electronically disfavors significant out‐of‐plane distortion, thereby providing both a structural and an electronic rationale for the suppression of 3D or interpenetrated network formation. High‐angle annular dark‐field scanning TEM (HAADF‐STEM; Figure S26) provides direct visual evidence for uniform incorporation of Pt^II^(PEt_3_)_2_ moieties into the framework. The metal content quantified by inductively coupled plasma mass spectrometry (ICP‐MS) confirmed efficient metal‐acetylide coordination (Table S2). AFM height profiles indicated nanosheet thicknesses of approximately 1.3 nm, corresponding to about four stacked layers of TTED‐Pt‐AF (Figure 2k). Thermogravimetric analysis (TGA) demonstrated that the TTED‐M‐AFs possess good thermal stability, remaining intact up to ∼230°C under a N_2_ atmosphere, with the coordinated M^II^(PEt_3_)_2_ units contributing to enhanced framework robustness (Figure S27).

### 2.2 Piezoelectric Polarization and Charge Transfer in TTED‐M‐AFs

**FIGURE 3:**
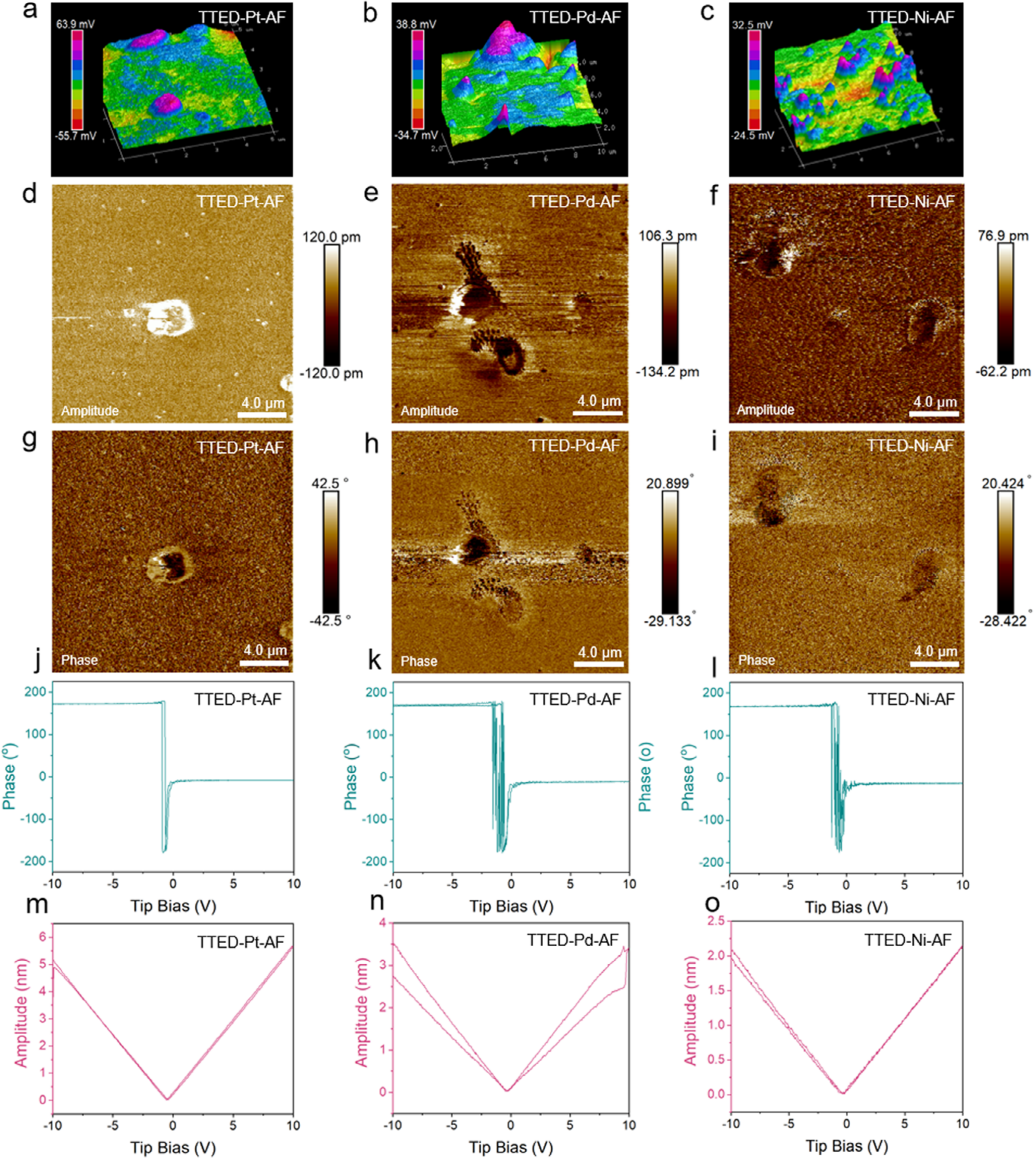
KPFM surface potential images of (a) TTED‐Pt‐AF, (b) TTED‐Pd‐AF, and (c) TTED‐Ni‐AF. PFM amplitude images of (d) TTED‐Pt‐AF, (e) TTED‐Pd‐AF, and (f) TTED‐Ni‐AF. Corresponding phase images of (g) TTED‐Pt‐AF, (h) TTED‐Pd‐AF, and (i) TTED‐Ni‐AF reveal polarization orientations with contrast between oppositely polarized domains. (j−l) The piezoelectric response, (m−o) hysteresis loops‐phase‐voltage and amplitude‐voltage butterfly loops demonstrate reversible and switchable domain behavior for (j, m) TTED‐Pt‐AF, (k, n) TTED‐Pd‐AF, and (l, o) TTED‐Ni‐AF, confirming robust piezoelectricity in the metal‐acetylide frameworks.

The piezoelectric polarization properties of the TTED‐M‐AFs and the metal‐free TTED‐GDY were quantitatively assessed using Kelvin probe force microscopy (KPFM) [41]. Three‐dimensional surface potential (SP) mappings (Figure 3a–c and Figure S28) revealed SP values of 63.9, 38.8, 32.5, and 12.9 mV for TTED‐Pt‐AF, TTED‐Pd‐AF, TTED‐Ni‐AF, and TTED‐GDY, respectively. The higher surface potential of TTED‐Pt‐AF indicates a stronger built‐in electric field and enhanced polarized charge density, reflecting its superior piezoelectric polarization and more efficient charge carrier transport. This enhancement is attributed to optimal 5*d_z_^2^*–2*p_z_* orbital overlap between the vacant d orbitals of Pt^II^ and the π‐system of the alkynyl ligands, enabled by the favorable spatial extension and energy matching of Pt 5d orbitals, which promotes extensive π‐electron delocalization and improves electron cloud mobility throughout the framework [42].

To further investigate the intrinsic piezoelectric response, we employed piezoresponse force microscopy (PFM) to characterize the local electromechanical activity via the converse piezoelectric effect, manifested as local volume expansion under an alternating electric field along the polarization axis [43]. The magnitude of this strain correlates directly with enhanced electron‐hole separation and charge transfer efficiency. PFM amplitude and phase images (Figure 3d–i) confirmed the presence of distinct piezoelectric polarization domains, with phase contrast revealing regions of opposite polarization orientation. Phase‐voltage and amplitude‐voltage hysteresis loops exhibited nearly 180° phase reversals and butterfly‐shaped amplitude responses (Figure 3j–o and Figure S28b,c), robustly confirming the presence of switchable piezoelectric domain structures in all three TTED‐M‐AFs. Notably, the maximum piezoresponse amplitude of TTED‐Pt‐AF (5.0 nm) exceeded those of TTED‐Pd‐AF (3.5 nm), TTED‐Ni‐AF (1.41 nm), and TTED‐GDY (0.43 nm) by factors of approximately 1.43, 3.55, and 11.63, respectively, underscoring its superior piezoelectric activity. TTED‐Pt‐AF exhibits the largest piezoelectric amplitude due to enhanced Pt^II^ d→π* backdonation, while TTED‐Pd‐AF displays pronounced ferroelectric hysteresis, indicative of switchable dipolar domains with optimal energy barriers for polarization reversal. This dichotomy highlights distinct structure–piezoresponse relationships across the isostructural series. The effective piezoelectric coefficients (*d_33_*), derived from the amplitude curves, were determined to be 35.0, 21.9, 12.5, and 2.67 pm V^−1^ for TTED‐Pt‐AF, TTED‐Pd‐AF, TTED‐Ni‐AF, and TTED‐GDY, respectively. As summarized in Table S3, the *d_33_* values of the TTED‐M‐AFs compare favorably with those of other reported 2D materials. Specifically, TTED‐Pt‐AF exhibits piezoelectric properties on par with or superior to those of MOFs, COFs, and g‐C_3_N_4_, while significantly outperforming transition metal dichalcogenides (TMDs) and MXenes. The enhanced polarization in TTED‐Pt‐AF likely stems from its higher mechanosensitivity, which facilitates greater framework deformation and rapid rearrangement of delocalized electrons under mechanical stimulus, thereby generating a stronger polarization field.

Complementary investigations of charge‐carrier dynamics and piezoelectric response were conducted by combining transient piezoelectric current measurements, electrochemical impedance spectroscopy (EIS), linear sweep voltammetry (LSV), and photoluminescence (PL) spectroscopy (Figure S29). Across all techniques, TTED‐Pt‐AF consistently outperformed the Pd^II^‐ and Ni^II^‐based analogues as well as the metal‐free control. Specifically, TTED‐Pt‐AF exhibits the highest piezoelectric current density, indicating generation of the greatest pool of free charges under mechanical stimulus; the smallest Nyquist semicircle diameter in EIS, reflecting the lowest charge‐transfer resistance; and the most quenched PL emission, indicating superior charge separation and transfer capabilities. Additionally, under continuous ultrasound stimulation, TTED‐Pt‐AF maintains a stable piezoelectric current over extended durations, whereas the Pd^II^‐ and Ni^II^‐based frameworks show noticeable signal decay, reflecting reduced long‐term stability of their piezoelectric responses. The electrocatalytic CO_2_ reduction performance of the TTED‐M‐AFs and TTED‐GDY was further evaluated by LSV in Ar‐ and CO_2_‐ saturated 0.5 m Na_2_SO_4_ electrolytes (Figure S29d,e). The current densities followed the order TTED‐GDY < TTED‐Ni‐AF < TTED‐Pd‐AF < TTED‐Pt‐AF, demonstrating the superior CO_2_ reduction activity of TTED‐Pt‐AF. Furthermore, for all TTED‐M‐AF electrodes, the current densities were lower in CO_2_‐saturated electrolyte than in Ar‐saturated electrolyte at the same potential (Figure S29e), confirming that these materials preferentially catalyze CO_2_ reduction over H_2_ evolution. Together, these results unequivocally establish TTED‐Pt‐AF as the leading candidate in this series for piezoelectric CO_2_ reduction, characterized by its exceptional piezoelecric activity, efficient charge carrier dynamics, and robust operational stability.

### 2.3 Piezo‐Catalytic CO_2_ Reduction Reaction (PCRR) Performance

**FIGURE 4:**
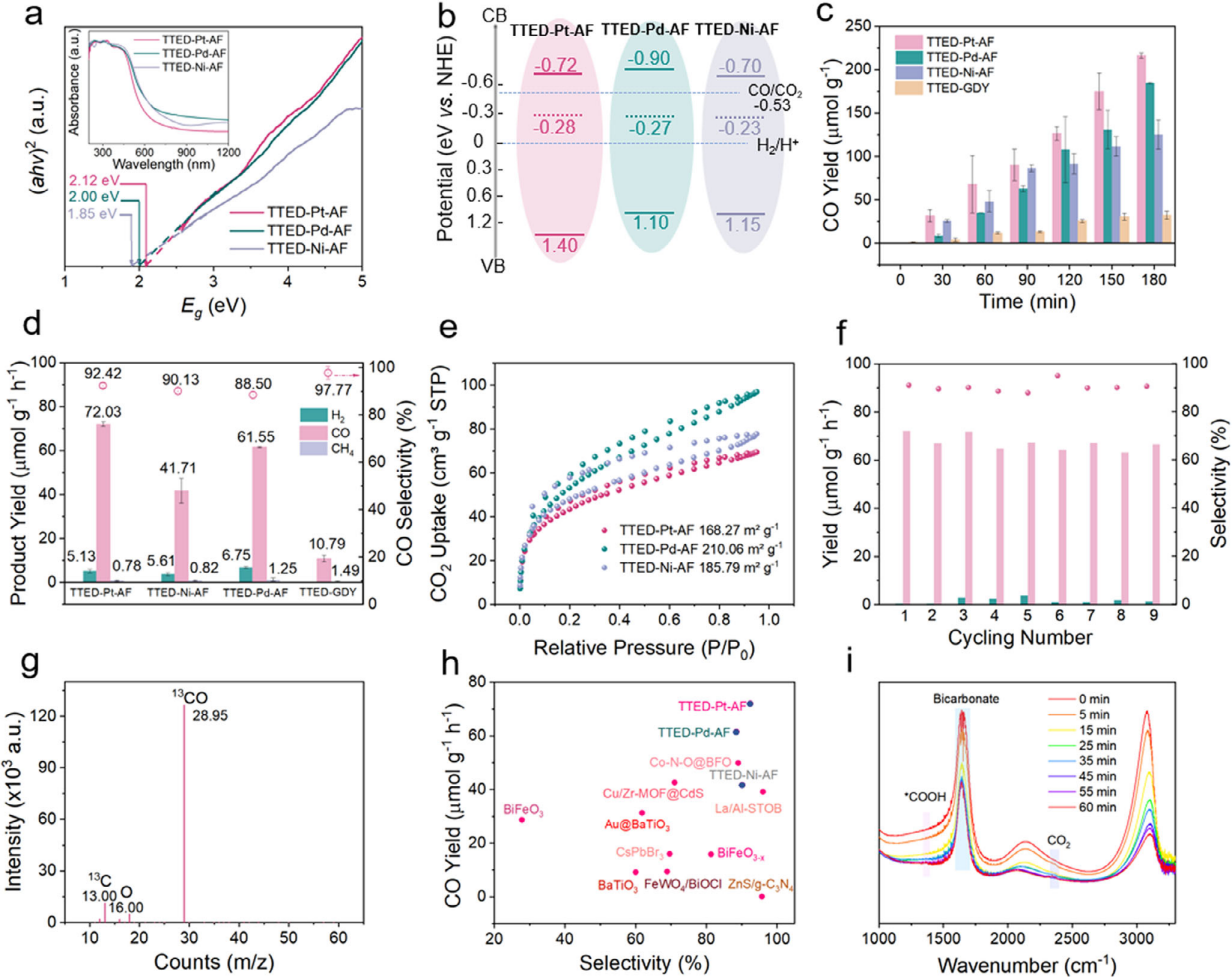
(a) Bandgap energies and the corresponding DRS spectra (inset) of TTED‐M‐AFs. (b) Schematic energy band structures of TTED‐M‐AFs. (c) Cumulative CO production over time and (d) production rates of CO, H_2,_ and CH_4_ via piezocatalysis for TTED‐M‐AFs and metal‐free TTED‐GDY. (e) CO_2_ adsorption isotherms measured at 197 K for TTED‐M‐AFs. (f) Stability test showing CO and H_2_ production rates over nine consecutive catalytic cycles using the TTED‐Pt‐AF/Na_2_SO_3_ system. (g) GC‐MS analysis of CO and CH_4_ products from isotopic labeling experiments conducted under a ^13^CO_2_ atmosphere. (h) Comparative piezoelectric CO_2_ reduction activity of TTED‐M‐AFs against previously reported piezoelectric materials (corresponding references cited in Refs. [2, 4, 46, 47, 48, 49, 50, 51, 52, 53]). (i) In situ DRIFTS monitoring of intermediates during piezoelectric CO_2_ reduction.

The electronic structures of the TTED‐M‐AFs were systematically investigated using ultraviolet‐visible diffuse reflectance spectra (UV–vis DRS), valence band X‐ray photoelectron spectroscopy (VB‐XPS), and Mott‐Schottky (M–S) analyses. UV–vis DRS revealed broad optical absorption spanning the visible to near‐infrared regions (inset, Figure 4a). Bandgap energies, determined from Tauc plots, were 1.85, 2.00, 2.21, and 2.51 eV for TTED‐Ni‐AF, TTED‐Pd‐AF, TTED‐Pt‐AF, and TTED‐GDY, respectively (Figure 4a and Figure S30), confirming that the incorporation of M^II^(PEt_3_)_2_ units facilitates electron excitation from the valence band (VB) to the conduction band (CB). Photographs of the corresponding samples are provided in Figure S31. The flat band potentials, measured vs. Ag/AgCl, were −0.52, −0.51, and −0.47 V (equivalent to −0.28, −0.27, and −0.23 V vs. NHE) for TTED‐Ni‐AF, TTED‐Pd‐AF, and TTED‐Pt‐AF, respectively (Figure S32a–c). VB‐XPS yielded valence band maxima of 1.15, 1.10, and 1.40 eV for the respective frameworks (Figure S32d–f). The derived CB potentials were −0.78, −0.90, and −0.72 eV, all more negative than the thermodynamic potential for CO_2_/CO reduction (−0.53 V vs. NHE), confirming the thermodynamic feasibility of CO_2_ reduction on these piezoelectric frameworks (Figure 4b).

The piezocatalytic CO_2_ reduction performance of TTED‐Pt‐AF, TTED‐Pd‐AF, TTED‐Ni‐AF, and TTED‐GDY is summarized in Figure 4c,d and Figure S33. Under optimized ultrasonic irradiation conditions (0.20 mmol Na_2_SO_3_ as a hole scavenger and a catalyst loading of 1 mg), all TTED‐M‐AFs facilitated the conversion of CO_2_ to CO, H_2_, and CH_4_. Notably, no other liquid products were detected by ^1^H NMR spectroscopy (Figure S34). The sulfite ions (SO_3_^2−^) are scavenged by piezogenerated holes and oxidized to sulfate (SO_4_^2−^), playing a crucial role in sustaining charge separation and facilitating the multi‐electron reduction of CO_2_ (Figure S35). Notably, TTED‐Pt‐AF achieved a CO production rate of 72.03 µmol g^−1^ h^−1^, surpassing those of TTED‐Pd‐AF (61.55 µmol g^−1^ h^−1^), TTED‐Ni‐AF (41.71 µmol g^−1^ h^−1^), and TTED‐GDY (10.79 µmol g^−1^ h^−1^) by factors of 1.17, 1.73, and 6.7, respectively, underscoring its superior piezoelectric response and charge transfer capability. Furthermore, TTED‐Pt‐AF exhibited the highest CO selectivity (92.42%), exceeding that of TTED‐Pd‐AF (88.50%) and TTED‐Ni‐AF (90.13%). CO_2_ adsorption isotherms measured at 195 K (Figure 4e) confirmed effective CO_2_ uptake by all TTED‐M‐AFs. Interestingly, TTED‐Pd‐AF (210.06 m^2^ g^−1^) and TTED‐Ni‐AF (185.79 m^2^ g^−1^) showed higher CO_2_ adsorption capacities than TTED‐Pt‐AF (168.27 m^2^ g^−1^), indicating that the enhanced PCRR efficiency of TTED‐Pt‐AF stems primarily from its superior piezoelectric polarization and charge separation efficiency, rather than from adsorption capacity.

The operational stability of TTED‐Pt‐AF was evaluated over eight consecutive catalytic cycles (Figure 4f), which confirmed the retention of both high PCRR activity and structural integrity (Figures S36–S39). Figure S37 reveals a positive shift in the P 2p and Pt 4f binding energies of TTED‐Pt‐AF post‐catalysis relative to the pristine state. This increase signifies electron density depletion from both the Pt^II^ center and phosphine ligands during CO_2_ reduction. Electron transfer from the Pt site through the conjugated framework activates and reduces CO_2_, thereby diminishing π‐backdonation to the phosphine, which reduces P electron density and elevates the P 2p binding energy. These XPS shifts provide direct evidence linking the active‐site electronic structure to catalytic electron donation. Isotope labelling experiments using ^13^CO_2_ (Figure 4g) verified that the carbon source of the reduction products originates exclusively from the supplied CO_2_. A comparative analysis with other reported 2D piezoelectric materials (Figure 4h) highlights the superior PCRR performance of the TTED‐M‐AFs, emphasizing their exceptional piezoelectric and catalytic properties.

To elucidate the reaction mechanism, in situ diffuse reflectance infrared Fourier transform spectroscopy (DRIFTS) was performed (Figure 4i). The temporal evolution of the band at 1646 cm^−1^, assigned to the C─O─C stretching vibration of bicarbonate species, showed a progressive decrease under ultrasonic vibration, indicating bicarbonate dissociation and concurrent CO_2_ activation. A concomitant increase in the signal for the COOH* intermediate was observed, supporting its role as a key reaction intermediate in the PCRR pathway [44]. The subsequent electron‐induced transformation of COOH* to CO validates the proposed reductive mechanism within these piezoactive frameworks [45].

### 2.4 Pressure‐Induced Deformation and Accelerated Charge Carrier Separation

**FIGURE 5:**
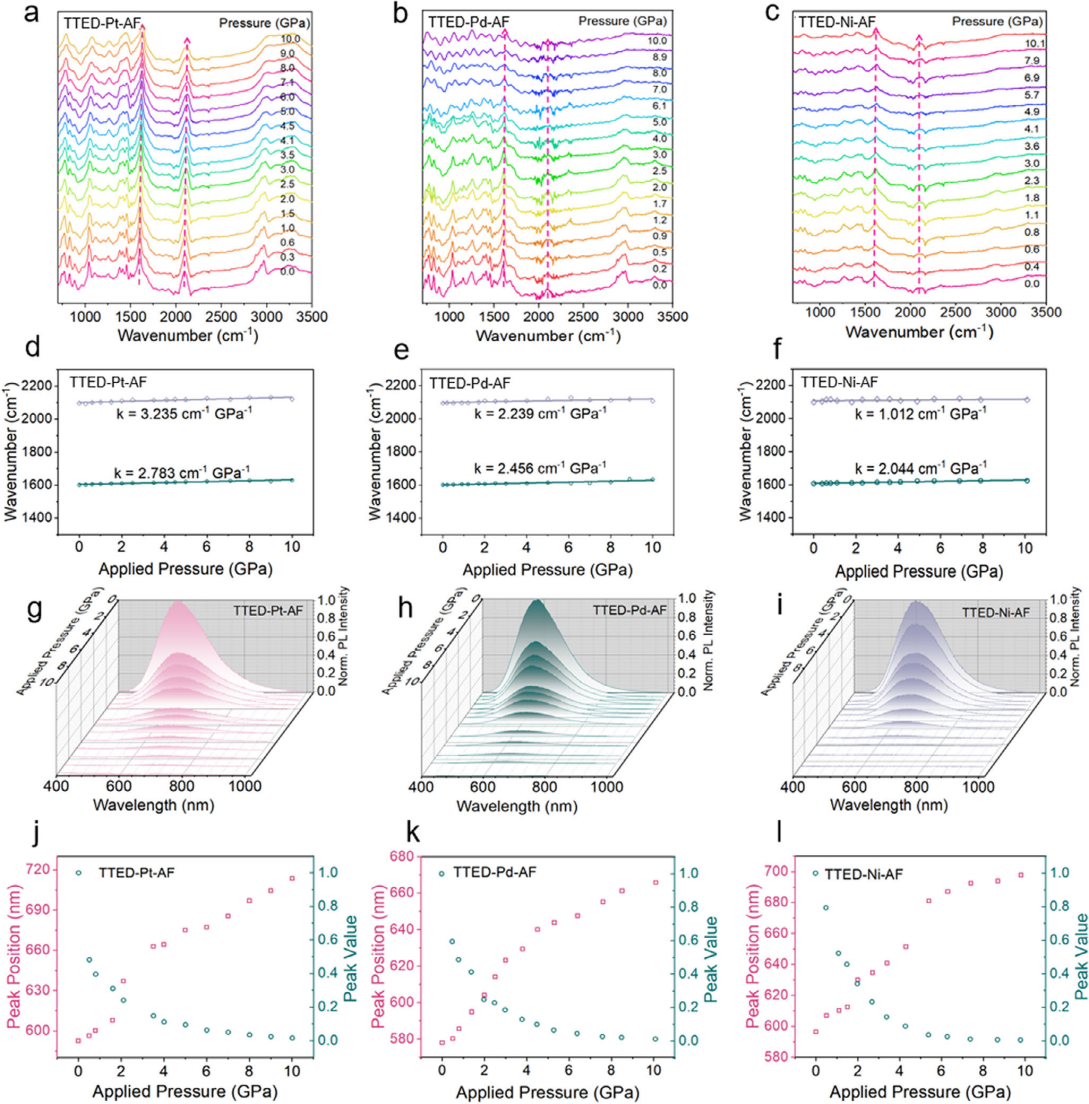
In situ high‐pressure FT‐IR spectra of (a) TTED‐Pt‐AF, (b) TTED‐Pd‐AF, and (c) TTED‐Ni‐AF under variable pressure conditions. Peak fitting analyses of the corresponding infrared absorption bands for (d) TTED‐Pt‐AF, (e) TTED‐Pd‐AF, and (f) TTED‐Ni‐AF. Pressure‐dependent PL spectra of (g) TTED‐Pt‐AF, (h) TTED‐Pd‐AF, and (i) TTED‐Ni‐AF. Pressure‐induced shifts in PL peak position (green dots) and intensity (red dots) for (j) TTED‐Pt‐AF, (k) TTED‐Pd‐AF, and (l) TTED‐Ni‐AF.

Mechanical deformation in piezocatalysts induces strong piezoelectric fields that promote charge carrier separation and migration [54]. The pressure‐dependent spectroscopic changes were monitored from ambient conditions up to 10 GPa. Notably, the most significant and mechanistically relevant evolution in vibrational and electronic structure occurs within the 0–2 GPa range, which directly corresponds to the cavitation pressures generated during ultrasonic irradiation. The trends observed at higher pressures (2–10 GPa) serve to amplify and confirm the intrinsic material properties that govern piezocatalytic performance under operating conditions. To elucidate pressure‐induced structural evolution, in situ high‐pressure FT‐IR spectroscopy was conducted on TTED‐Pt‐AF, TTED‐Pd‐AF, TTED‐Ni‐AF, and the metal‐free TTED‐GDY control (Figure 5a–c). Characteristic vibrational modes associated with alkyne bonds and benzene rings exhibited systematic blue shifts with increasing pressure. For TTED‐Pt‐AF, the alkyne stretching frequency shifted from 2096 to 2121 cm^−1^ as pressure increased from ambient to 10 GPa. Under identical conditions, TTED‐Pd‐AF, TTED‐Ni‐AF, and TTED‐GDY exhibited smaller shifts from 2094 to 2108 cm^−1^, 2108 to 2111 cm^−1^, and 2100 to 2110 cm^−1^, respectively. Similarly, benzene ring vibrations blue‐shifted from 1601 to 1630 cm^−1^ for TTED‐Pt‐AF, while TTED‐Pd‐AF, TTED‐Ni‐AF, and TTED‐GDY shifted from 1600 to 1620 cm^−1^, 1610 to 1624 cm^−1^, and 1604 to 1618 cm^−1^, respectively. Quantitative analysis revealed that the alkyne blue‐shift rates followed the order: TTED‐Pt‐AF (3.235 cm^−1^ GPa^−1^) > TTED‐Pd‐AF (2.239 cm^−1^ GPa^−1^) > TTED‐Ni‐AF (1.102 cm^−1^ GPa^−1^) > TTED‐GDY (0.625 cm^−1^ GPa^−1^) (Figure 5d–f and Figure S40). A parallel trend was observed for benzene ring blue‐shift rates: TTED‐Pt‐AF (2.783 cm^−1^ GPa^−1^) > TTED‐Pd‐AF (2.456 cm^−1^ GPa^−1^) > TTED‐Ni‐AF (2.044 cm^−1^ GPa^−1^) > TTED‐GDY (1.345 cm^−1^ GPa^−1^), indicating enhanced vibrational sensitivity to mechanical stress imparted by the M^II^(PEt_3_)_2_ moieties. The pronounced blue shifts in TTED‐Pt‐AF are attributed to intensified intermolecular repulsive interactions under compression, which reduce interlayer spacing and amplify structural coupling [55]. These results signify the heightened susceptibility of the TTED‐Pt‐AF framework to pressure‐induced asymmetric structural reorganization, driven by the Pt^II^(PEt_3_)_2_ units, which generate stronger local dipole moments and contribute to the observed infrared spectral shifts [56]. Correspondingly, TTED‐Pt‐AF demonstrated superior piezocatalytic performance relative to its Pd^II^‐ and Ni^II^‐based analogues, with the ─C≡C─M(PEt_3_)_2_─C≡C─ vibrational motifs playing a pivotal role in pressure‐enhanced piezoelectric activity.

In situ high‐pressure PL spectroscopy was employed to probe charge recombination dynamics under compression [57]. Upon increasing pressure from ambient to 10 GPa, TTED‐M‐AFs exhibited piezochromic behavior, transitioning in color from yellow to red and finally to colorless (Figures S41–S43), while TTED‐GDY changed from red to colorless (Figure S44), highlighting their potential as pressure‐responsive piezochromic materials. Concurrently, PL peak intensities decreased substantially and exhibited significant red shifts with increasing pressure (Figure 5g–i and Figure S45), indicating suppressed charge recombination. The observed red shift is primarily attributed to pressure‐enhanced π–π stacking interactions [58]. Among the series, TTED‐Pt‐AF displayed the most pronounced red shift and the greatest degree of fluorescence quenching, reflecting enhanced electron delocalization and substantial structural modulation, which collectively contribute to more effective suppression of charge recombination compared to TTED‐Pd‐AF, TTED‐Ni‐AF, and TTED‐GDY (Figure 5j–l).

Under compressive stress of 2 GPa, the tetraphenyl π‐columns undergo considerable conformational distortion, promoting enhanced π‐orbital overlap and electronic delocalization. Simultaneously, the initially linear ethynyl ─C≡C─ linkers experience bending due to steric compression, generating localized dipole moments along these bonds. This results in macroscopic piezoelectric polarization oriented orthogonally to the stacking direction [59]. The resulting internal electric field directs the migration of charge carriers toward the strained ethynyl moieties, where the electron‐deficient bent ─C≡C─ bonds function as efficient charge storage sites, stabilized by resonance between the aromatic core and the distorted metal‐carbon units [60, 61]. This mechanistic framework aligns with the observed PL red shifts, which reflect the stabilization of charge‐transfer excited states under pressure, and affirms the direct correlation between pressure‐modified molecular architecture and enhanced piezoelectric charge storage capability.

### 2.5 Theoretical Study of the Piezoelectric CO_2_ Reduction Mechanism

DFT calculations were conducted using Ar─C≡C─M(PEt_3_)_2_─C≡C─Ar model (Ar represents dibenzog, pchrysene). The calculated *cis* configuration arises from the use of a simplified, flexible ligand set to clarify the reaction mechanism. This choice doesn't restrict the applicability of the proposed electronic modulation, which is expected to extend to the *trans* centers present in the actual system, because the modulation operates on the intrinsic and universal electronic properties of the ─C≡C─M─C≡C─ block. to elucidate the catalytic mechanism of CO_2_‐to‐CO conversion over TTED‐M‐AFs under ultrasonic vibration, with a focus on CO_2_ adsorption configurations and charge density differences change at an applied pressure of 2 GPa. The calculated CO_2_ adsorption energies on the ─C≡C─M(PEt_3_)_2_─C≡C─ linkages were significantly more negative than those on the tetraphenyl π‐column centers, confirming that the metal‐bis(acetylide) moieties serve as the preferential adsorption and catalytic sites, facilitated by strong π‐*d*‐π interactions involving the 16 valence electrons of the transition M^II^ ion (Figure S46). Among the series, the ─C≡C─Pt(PEt_3_)_2_─C≡C− unit exhibited the strongest CO_2_ adsorption interaction (*E_ads_* = −1.317 eV) under pressure, surpassing the Pd^II^ (−1.02 eV) and Ni^II^ (−0.975 eV) analogues. In contrast, the adsorption energy for the Pt^II^‐based unit under ambient conditions was notably less negative (−0.682 eV), highlighting the role of pressure in enhancing CO_2_ binding (Figure S46b). The charge distribution was investigated using the electrostatic potential surface (ESP) of TTED‐M‐AFs. Particularly, the electron‐rich regions are mainly distributed around the ─C≡C─M(PEt_3_)_2_─C≡C─ sites, contributing to the combination with CO_2_ molecules. In addition, the ESP mappings also indicated that these TTED‐M‐AFs could build an internal polarization field, further enhancing the catalytic performance (Figure S47).

**FIGURE 6:**
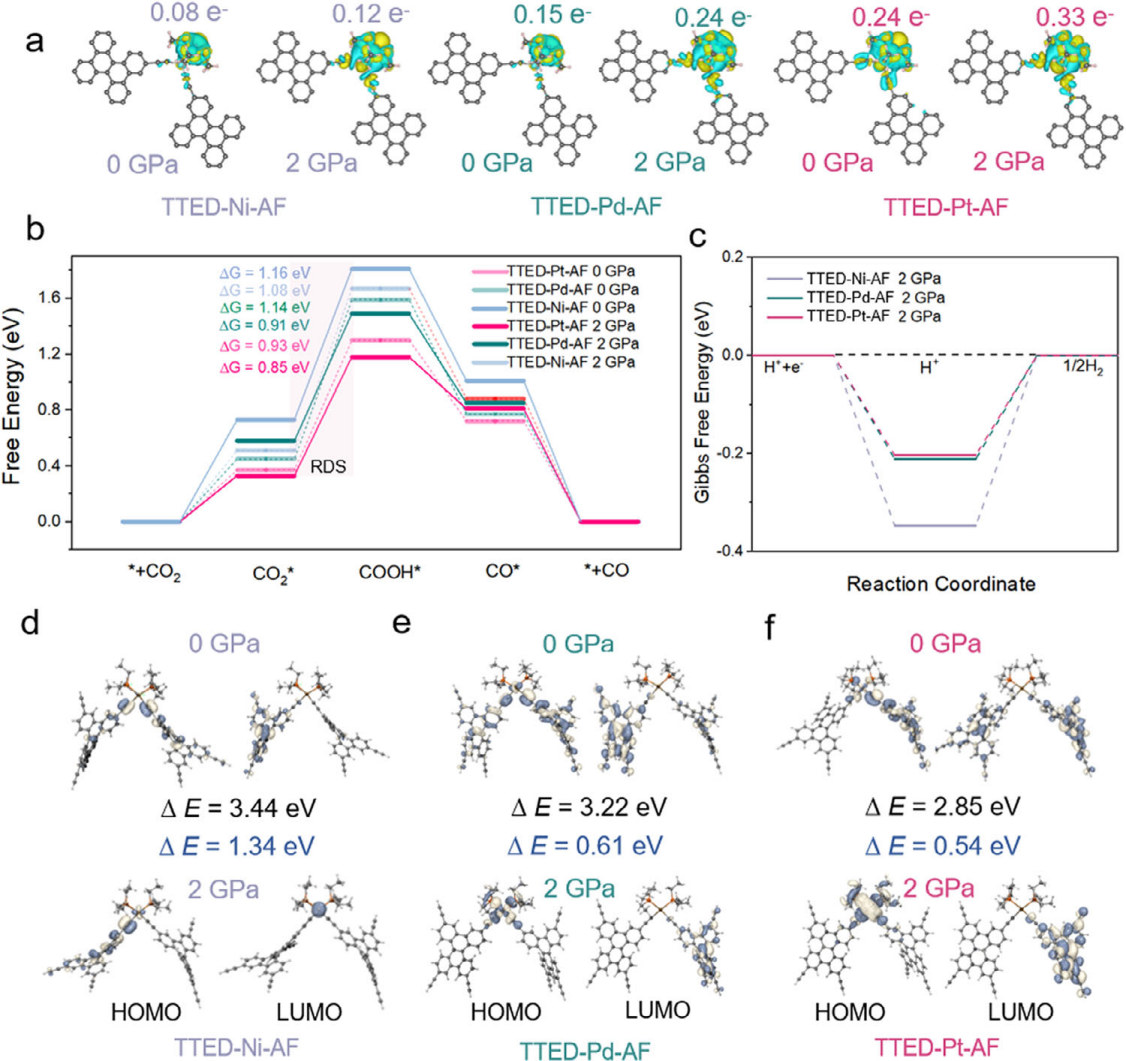
(a) Isosurfaces of charge density differences illustrating CO_2_ adsorption on the ─C≡C─M(PEt_3_)_2_─C≡C─ moiety, where M = Pt, Pd, and Ni, and the corresponding Barder charge number CO_2_ adsorption under ambient and 2 GPa pressure. Gibbs free energy profiles for (b) CO and (c) H_2_ generation pathways on TTED‐M‐AFs under 2 GPa pressure. (d–f) Frontier molecular orbitals (HOMO and LUMO) of TTED‐M‐AFs calculated with and without applied 2 GPa pressure.

The charge density difference analysis at 0 GPa shows relatively weak electronic coupling between CO_2_ and the metal‐bis(acetylide) moiety in TTED‐Ni‐AF and TTED‐Pd‐AF, with spatially separated electron densities indicating a substantial energy barrier (Figure 6a). In contrast, TTED‐Pt‐AF exhibits pronounced electron accumulation near the ─C≡C─Pt(PEt_3_)_2_─C≡C─ centers, consistent with stronger CO_2_ binding. Bader charge analysis quantifies electron transfer from the metal‐bis(acetylide) sites to CO_2_ as approximately 0.24, 0.15, and 0.08 e^−1^ for Pt^II^, Pd^II^, and Ni^II^, respectively, underscoring the strongest electrostatic interaction in TTED‐Pt‐AF under ambient conditions. Under 2 GPa pressure, electronic coupling between CO_2_ and the ─C≡C─M(PEt_3_)_2_─C≡C─ moiety becomes markedly enhanced, with more pronounced electron accumulation around Pt‐bis(acetylide) centers. Bader analysis indicates greater electron transfer to CO_2_ of about 0.33, 0.24, and 0.12 e^−1^ for Pt^II^, Pd^II^, and Ni^II^, respectively, confirming the strongest electrostatic interaction in TTED‐Pt‐AF with pressure. The carbon atom of CO_2_ acts as the principal electron acceptor, populating the antibonding 2π_u_ orbitals under 2 GPa, which correlates with pronounced bending of the O─C─O angle (168.66°, 172.70°, 172.97°) and elongation of C─O bonds (1.21, 1.19,1.19 Å) for Pt^II^‐, Pd^II^‐, and Ni^II^‐based frameworks, respectively (Figures S46 and S48). Moreover, pressure‐induced asymmetry in the Pt^II^‐bis(acetylide) units further promotes electron transfer [62]. Reference energy analyses indicate that piezoelectric polarization, driven by ultrasonic vibration, plays a critical role in activating key intermediates and enhancing CO_2_ reduction under ambient conditions [63].

CO_2_ reduction pathways were evaluated by computing reaction energy profiles at ambient and 2 GPa (Figure 6b and Figure S49). Activation initiates with CO_2_ adsorption at the ─C≡C─M(PEt_3_)_2_─C≡C− sites to form adsorbed *CO_2_, followed by a proton‐electron transfer to form *COOH, identified as the rate‐determining step. Under 2 GPa pressure, TTED‐Pt‐AF exhibits the lowest barrier for *COOH formation (0.85 eV) relative to TTED‐Pd‐AF (0.91 eV) and TTED‐Ni‐AF (1.08 eV), demonstrating superior CO_2_ hydrogenation efficiency for Pt^II^‐bis(acetylide) moieties under pressure. Under ambient pressure, the barriers are higher (0.94, 1.14, and 1.16 eV for TTED‐Pt‐AF, TTED‐Pd‐AF, TTED‐Ni‐AF, respectively), highlighting the synergistic role of piezoelectric polarization in activating key intermediates and enhancing CO_2_ reduction [48].

H_2_ evolution reaction (HER) pathways were also considered (Figure 6c). The calculated rate‐determining step barriers are 0.347, 0.211, and 0.203 eV for TTED‐Ni‐AF, TTED‐Pd‐AF, and TTED‐Pt‐AF, respectively, aligning with the experimentally observed H_2_ production trends. The high CO selectivity of TTED‐Pt‐AF (92.4%), despite its intrinsically low HER barrier, is rationalized by a competitive kinetic model under reaction conditions. In CO_2_ atmosphere, the ─C≡C─Pt(PEt_3_)_2_─C≡C− active sites are preferentially occupied by CO_2_ due to its strong adsorption (*E_ads_* = −1.317 eV), effectively blocking proton access and suppressing the HER pathway. The piezoelectric field then stabilizes the *COOH intermediate, directing electron flux toward CO production. This model is corroborated by control experiments under Ar: in the absence of CO_2_, protons gain access to the sites, yielding high H_2_ evolution rates of 3131.42, 2759.27, and 2381.30 µmol g^−1^ h^−1^ for TTED‐Pt‐AF, TTED‐Pd‐AF, and TTED‐Ni‐AF, respectively (Figure S50). Thus, selectivity is governed by competitive adsorption under operating conditions, not solely by intrinsic kinetic barriers calculated in vacuum.

To clarify the electron transfer mechanism during PCRR, the frontier molecular orbitals, specifically the highest occupied molecular orbital (HOMO) and the lowest unoccupied molecular orbital (LUMO), were computed at 0 and 2 GPa (Figure 6d–f). Under ambient conditions, both HOMO and LUMO were predominantly localized on the TTED ligand with substantial spatial overlap, which is unfavorable for charge separation. The HOMO–LUMO energy gaps were 2.85, 3.32, and 3.44 eV for TTED‐Pt‐AF, TTED‐Pd‐AF, and TTED‐Ni‐AF, respectively, modulated by bandgap renormalization effects arising from layer stacking [64, 65]. Under 2 Gpa compression, the HOMOs of TTED‐Pt‐AF and TTED‐Pd‐AF shifted toward the ─C≡C─M(PEt_3_)_2_─C≡C─ cores, while the LUMOs localized on the tetraphenyl π‐columns, favoring metal‐to‐ligand charge transfer (MLCT) and stabilizing charge‐separated states [66]. In contrast, TTED‐Ni‐AF retained HOMO localization on the TTED ligands and LUMO on the Ni sites, with substantial orbital overlap promoting ligand‐to‐metal charge transfer (LMCT) and increasing charge recombination probability. Electron transfer from the ─C≡C─M(PEt_3_)_2_─C≡C─ units to CO_2_ is essential for catalytic efficacy. In TTED‐Ni‐AF, this occurs predominantly via LMCT, whereas TTED‐Pt‐AF and TTED‐Pd‐AF enable direct electron donation from metal‐centered HOMOs to CO_2_, with the tetraphenyl π‐columns assisting in electron stabilization and potential reinjection during catalysis. Applied pressure significantly narrows the HOMO‐LUMO energy gaps (*ΔE*), with TTED‐Pt‐AF exhibiting the smallest gap and highest electronic conductivity. Under pressure, HOMO energies rise markedly while LUMO energies remain relatively stable, indicating selective mechanical modulation of the electronic structure (Table S4). Collectively, these theoretical results demonstrate that piezoelectric polarization under mechanical stress promotes favorable orbital rearrangements, enhances charge separation, and stabilizes key reaction intermediates. Among the series, TTED‐Pt‐AF exhibits the most efficient electronic structure and mechanistic pathway for piezocatalytic CO_2_ reduction.

## 3 Conclusion

In summary, we have designed and systematically investigated a series of 2D metal‐acetylide frameworks (TTED‐M‐AFs; M = Ni, Pd, Pt) for efficient piezocatalytic CO_2_ reduction. Among them, the Pt^II^‐based framework, TTED‐Pt‐AF, exhibits exceptional performance, delivering a CO production rate of 72.03 µmol g^−1^ h^−1^ with 92.42% selectivity under ultrasonic excitation, significantly surpassing its Pd^II^ and Ni^II^ analogues. This superior activity originates from the pronounced structural asymmetry and enhanced electronic polarization induced by the Pt^II^(PEt_3_)_2_ moieties under mechanical stress, which activates the ─C≡C─Pt(PEt_3_)_2_─C≡C─ motifs as highly effective catalytic sites. The superior PCRR activity of TTED‐Pt‐AF, despite its lower specific surface area and CO_2_ adsorption capacity, can be unequivocally attributed to its intrinsically stronger piezoelectric response. PFM measurements directly quantify this enhanced piezoelectricity, with TTED‐Pt‐AF exhibiting a d_33_ coefficient of 35.0 pm V^−1^, substantially higher than its Pd and Ni analogues. This stronger piezoelectric field drives superior charge carrier dynamics, as evidenced by its higher transient piezocurrent, lower charge‐transfer resistance, more efficient PL quenching, and enhanced electron delocalization. Collectively, multiple independent lines of evidence converge on a complete and coherent mechanistic picture: stronger piezoelectricity enables more efficient charge generation and separation, which facilitates enhanced charge transfer to adsorbed CO_2_, ultimately leading to higher catalytic activity. This causal chain explains why TTED‐Pt‐AF outperforms its analogues regardless of its physical specific surface area. It is worth emphasizing that the piezoelectric behavior and piezocatalytic performance of TTED‑M‑AFs do not depend on long‑range periodic order. Even with low crystallinity, local asymmetry and polarizability, arising from the ─C≡C─M(PEt_3_)_2_─C≡C─ motifs and their d‑π conjugation with the tetraphenylene backbone, are sufficient to generate a piezoelectric field under mechanical stress. The observed differences in piezocatalytic activity across Pt, Pd, and Ni analogues correlate with the intrinsic electronic structure of the metal centers (d‑orbital energy and spatial extension) rather than with the degree of crystallinity. Thus, the structure‑property‑performance relationship remains valid even when the materials are described as poorly crystalline or locally ordered layered frameworks.

Mechanistic studies reveal that electron transfer proceeds predominantly from metal‐centered HOMOs to chemisorbed CO_2_, while the tetraphenyl‐based π‐conjugated columns, with their low‐lying LUMOs, stabilize transient electrons and facilitate electron back‐donation to metal centers during catalytic turnover. The integration of Pt^II^(PEt_3_)_2_ units into the organic scaffold creates a remarkable electronic synergy, characterized by strong d–π conjugation that results in a highly delocalized and polarizable electronic structure. This configuration leads to a high piezoelectric coefficient due to facile electron cloud polarization under mechanical perturbation, generating a strong built‐in electric field that drives efficient charge separation and transfer while simultaneously lowering the energy barrier for CO_2_ adsorption and activation. Moreover, the downshift of the *d*‐band center induced by Pt incorporation thermodynamically suppresses competing side reactions, therebyenhancing CO selectivity. Building on our earlier work with a Ni^II^‐acetylide framework based on a hexaethynyltriphenylene ligand [30], the present study underscores the essential role of synergistic organic‐metal interactions in optimizing piezocatalytic performance. This study establishes both a validated mechanistic framework and a rational design strategy for mechano‐catalysis. By systematically varying the metal center (Pt, Pd, Ni) within an isostructural series of 2D metal‐acetylide frameworks, we directly demonstrate how tuning the strength of d−π conjugation dictates piezoelectric polarization. This, in turn, governs charge carrier dynamics and ultimately determines CO_2_ reduction activity. Overall, this work establishes metal‐acetylide frameworks as a highly promising class of 2D piezocatalysts and provides fundamental design principles for developing advanced piezo‐responsive materials, opening new avenues for mechano‐energy conversion and sustainable CO_2_ valorization.

## Author Contributions

**Mude Zhu**: conceptualization, methodology, investigation, writing – original draft, writing – review and editing, data curation, formal analysis. **Yingtang Zhou**: software, formal analysis, writing – review and editing. **Kai Wang**: methodology, writing – review and editing, validation, formal analysis. **Xiaoyun Fan**: funding acquisition, writing – review and editing, methodology. **Yang Ding**: methodology, writing – review and editing, resources. **Wai – Yeung Wong**: conceptualization, supervision, funding acquisition. **Linli Xu**: project administration, visualization, writing – review and editing, supervision, funding acquisition, resources.

## Funding

This work was financially supported by the Hong Kong Research Grants Council (PolyU 25301524; Young Collaborative Research Grant C5001‐24), Shenzhen Science and Technology Program (JCYJ20250604185428038), Guangdong Provincial Natural Science Foundation‐General Project (2024A1515010422), and PolyU (CE2N, CDB5, CE35, CE01, CEE7, CDDA). The financial support from the RGC Senior Research Fellowship Scheme (SRFS2021‐5S01), the Hong Kong Research Grants Council (PolyU 15307321), Research Institute for Smart Energy (CDAQ), Research Centre for Carbon‐Strategic Catalysis (CE41), and Miss Clarea Au for the Endowed Professorship in Energy (847S). The National Natural Science Foundation of China (52272112).

## Conflicts of Interest

The authors declare no conflicts of interest.

## Supporting information




**Supporting File**: advs75849‐sup‐0001‐SuppMat.docx.

## Data Availability

The data that support the findings of this study are available in the supplementary material of this article.
