# Supplementary material for: Tailored D‐π Conjugation Boosts Piezocatalytic CO2 Reduction in a Platinum(II)‐Acetylide Framework
Source: Adv Sci (Weinh). 2026 May 28;13(47):e75849. doi: 10.1002/advs.75849 (PMC13335834; doi:10.1002/advs.75849)
Supplement: Supplementary file 1 [file ADVS-13-e75849-s001.docx]

**Supporting Information**

**Tailored d-π Conjugation Boosts Piezocatalytic CO_2_ Reduction in a Platinum(II)-Acetylide Framework**

Mude Zhu,^[a]^ Yingtang Zhou,*^[b]^ Kai Wang,^[c]^ Xiaoyun Fan,^[d]^ Yang Ding,^[e]^ Wai-Yeung Wong,^[a]^ and Linli Xu*^[a]^

[a] Department of Applied Biology and Chemical Technology and Research Institute for Smart Energy, The Hong Kong Polytechnic University, Hung Hom, Kowloon, Hong Kong SAR, P. R. China.

[b] Marine Science and Technology College, Zhejiang Ocean University, 316004, Zhoushan, P. R. China.

[c] School of Physics Science and Information Technology, Liaocheng University, Liaocheng, 252000, P. R. China.

[d] College of Environment and Climate, Guangdong Provincial Key Laboratory of Environmental Pollution and Health, Jinan University, Guangzhou, 510632, P. R. China.

[e] Engineering Research Center for Semiconductor Integrated Technology, Institute of Semiconductors, Chinese Academy of Sciences, Beijing 100083, P. R. China

E-mail: [linli.xu@polyu.edu.hk](mailto:linli.xu@polyu.edu.hk) (L. L. X), [zhouyingtang@zjou.edu.cn](mailto:zhouyingtang@zjou.edu.cn) (Y. T. Z)

**Contents**

[1 General Information	5](#_Toc30043)

[1.1 Chemicals	5](#_Toc14167)

[1.2 Characterization techniques	6](#_Toc10395)

[2 Experimental Sections	7](#_Toc536)

[2.1 Synthesis of](#_Toc13561) *[trans](#_Toc13561)*[-Ni(PEt](#_Toc13561)_[3](#_Toc13561)_[)](#_Toc13561)_[2](#_Toc13561)_[Cl](#_Toc13561)_[2](#_Toc13561)_	[7](#_Toc13561)

[2.2 Synthesis of](#_Toc18621) *[trans](#_Toc18621)*[-Pd(PEt](#_Toc18621)_[3](#_Toc18621)_[)](#_Toc18621)_[2](#_Toc18621)_[Cl](#_Toc18621)_[2](#_Toc18621)_	[7](#_Toc18621)

[2.3 Synthesis of](#_Toc2278) *[trans](#_Toc2278)*[-Pt(PEt](#_Toc2278)_[3](#_Toc2278)_[)](#_Toc2278)_[2](#_Toc2278)_[Cl](#_Toc2278)_[2](#_Toc2278)_	[8](#_Toc2278)

[2.4 Synthesis of TTED-M-AFs	8](#_Toc5111)

[2.5 Synthesis of TTED-GDY	9](#_Toc5713)

[2.6 In situ high-pressure PL and IR absorption spectroscopies	10](#_Toc21006)

[2.7 Preparation of nanosheets for AFM characterization	10](#_Toc3626)

[2.8 PFM and KPFM measurements	10](#_Toc29980)

[2.9 Carrier transfer behavior assessment	11](#_Toc10224)

[2.10 Calculation of energy bands for TTED-M-AFs	11](#_Toc17147)

[2.11 Calculation of CO production and selectivity	12](#_Toc15154)

[2.12 DFT simulations	12](#_Toc2744)

[3 Supplementary Figures and Tables	14](#_Toc30486)

[Figure S1. (a)](#_Toc22233) ^[1](#_Toc22233)^[H, (b)](#_Toc22233) ^[13](#_Toc22233)^[C and (c)](#_Toc22233) ^[31](#_Toc22233)^[P NMR spectra of](#_Toc22233) *[trans](#_Toc22233)*[-Pd(PEt](#_Toc22233)_[3](#_Toc22233)_[)](#_Toc22233)_[2](#_Toc22233)_[Cl](#_Toc22233)_[2](#_Toc22233)_[.	14](#_Toc22233)

[Figure S2. (a)](#_Toc26606) ^[1](#_Toc26606)^[H and (b)](#_Toc26606) ^[13](#_Toc26606)^[C NMR spectra of TTED ligand.	15](#_Toc26606)

[Figure S3. Simulated PXRD patterns of TTED-Pt-AF for AA and AB stacking configurations.	16](#_Toc1731)

[Figure S4. (a) Simulated PXRD patterns of TTED-Pd-AF for AA and AB stacking configurations. (b) Experimental PXRD pattern of TTED-Pd-AF (red) overlaid with the profile calculated from Pawley refinement (blue), along with the difference curve (residual, yellow), compared to the simulated pattern derived from the optimized structural model (green). Reflection positions are indicated by vertical tick marks. (c) Approximate unit cell parameters from Pawley refinement of TTED-Pd-AF.	17](#_Toc19065)

[Figure S5. (a) Simulated PXRD patterns of TTED-Ni-AF for AA and AB stacking configurations. (b) Experimental PXRD pattern of TTED-Ni-AF (red), Pawly refinement simulated profile (blue), residual difference curve (yellow), and the simulated pattern from the optimized structural model (green). Reflection positions are denoted by vertical tick marks. (c) Approximate unit cell parameters from Pawley refinement of TTED-Ni-AF.	18](#_Toc7010)

[Figure S6. HR-TEM images of TTED-Pd-AF: (a) lattice fringes corresponding to the (1 0 2) crystallographic plane and (b) interlayer spacing resolved within the stacked framework.	19](#_Toc7802)

[Figure S7. HR-TEM images of TTED-Ni-AF: (a) lattice fringes corresponding to the (0 0 2) crystallographic plane and (b) interlayer spacing resolved within the stacked framework.	20](#_Toc5609)

[Figure S8. HR-TEM images of TTED-Pt-AF. (a) Lateral view highlighting the crystallographic plane (2 0 1), (b) and (c) magnified HR-TEM images of TTED-Pt-AF, showing local short-range order and a local pore‑like features.	21](#_Toc18149)

[Figure S9. Raman spectra of TTED-M-AFs.	22](#_Toc25029)

[Figure S10. Solid-state](#_Toc27799) ^[13](#_Toc27799)^[C CP-MAS NMR spectra of (a) TTED-Pd-AF and (b) TTED-Ni-AF.	23](#_Toc27799)

[Figure S11. Solid-state](#_Toc19194) ^[31](#_Toc19194)^[P NMR spectrum of TTED-Pt-AF.	24](#_Toc19194)

[Figure S12. N](#_Toc16312)_[2](#_Toc16312)_ [adsorption-desorption isotherms along with corresponding pore size distributions for (a) TTED-Pt-AF, (b) TTED-Pd-AF, and (c) TTED-Ni-AF. The isotherms display characteristic physisorption behavior, and the derived pore size distributions reveal narrow microporous channels centered around 0.78 nm across all samples.	25](#_Toc16312)

[Figure S13. Bulk powder volume of TTED-M-AFs (M = Pt, Pd, Ni), illustrating the characteristic low-density morphology of layered frameworks.	26](#_Toc19578)

[Figure S14. Calculated average pore diameter distribution for TTED-Pt-AF based on structural model.	27](#_Toc21810)

[Figure S15. Calculated average pore diameter distribution for TTED-Pd-AF based on the structural model.	28](#_Toc7744)

[Figure S16. Calculated average pore diameter distribution for TTED-Ni-AF based on the structural model.	29](#_Toc2279)

Figure S17. (a) Survey XPS spectra of TTED-M-AFs (M = Pt, Pd, Ni). (b) High-resolution P 2p core-level XPS spectrum of TTED-Pt-AF. (c) High-resolution Pt 4f core-level XPS spectrum of TTED-Pt-AF. (d) High-resolution C 1s core-level XPS spectrum of TTED-Ni-AF. (e) P 2p core-level XPS spectrum of TTED-Ni-AF. (f) Ni 2p core-level XPS spectrum of TTED-Ni-AF. (g) C 1s core-level XPS spectrum of TTED-Pd-AF. (h) P 2p core-level XPS spectrum of TTED-Pd-AF. (i) Pd 3d core-level XPS spectrum of TTED-Pd-AF....................30

[Figure S18. (a) Survey XPS spectra of TTED-GDY. (b) High-resolution C 1s core-level XPS spectrum of TTED-GDY.	31](#_Toc26929)

[Figure S19. Linear fitting of Pt white-line intensity for standard samples, establishing a calibration curve used to determine the oxidation state of Pt in TTED-Pt-AF.	32](#_Toc21128)

[Figure S20. EXAFS analysis at the Pt](#_Toc30479) *[L](#_Toc30479)_[3](#_Toc30479)_*[-edge for TTED-Pt-AF and reference materials. Fourier-transformed EXAFS spectra (](#_Toc30479)*[R](#_Toc30479)*[-space, magnitude) with experimental data (points) and theoretical fits (lines) for (a) TTED-Pt-AF, (b) PtO](#_Toc30479)_[2](#_Toc30479)_[, and (c) Pt foil. (d)](#_Toc30479) *[k](#_Toc30479)^[3](#_Toc30479)^*[-weighting EXAFS spectra (](#_Toc30479)*[k](#_Toc30479)*[-space) of Pt foil, PtO](#_Toc30479)_[2](#_Toc30479)_[, and TTED-Pt-AF. Corresponding](#_Toc30479) *[k](#_Toc30479)*[-space fitting curves (lines) overlaid on experimental data (points) for (e) TTED-Pt-AF, (f) PtO](#_Toc30479)_[2](#_Toc30479)_[, and (g) Pt foil. 3D wavelet transform plots of the](#_Toc30479) *[k](#_Toc30479)^[3](#_Toc30479)^*[-weighted Pt](#_Toc30479) *[L](#_Toc30479)_[3](#_Toc30479)_*[-edge EXAFS spectra for (h) Pt foil and (i) PtO](#_Toc30479)_[2](#_Toc30479)_[, respectively.	33](#_Toc30479)

[Figure S21. SEM images of TTED-Pt-AF, accompanied by EDS elemental mapping for C, Pt, and P.	34](#_Toc10817)

[Figure S22. SEM images of TTED-Pd-AF, accompanied by EDS elemental mapping for C, Pd, and P.	35](#_Toc9992)

[Figure S23. SEM images of TTED-Ni-AF, accompanied by EDS elemental mapping for C, Ni, and P.	36](#_Toc17294)

[Figure S24. SEM images of TTED-GDY, accompanied by EDS elemental mapping for C.	37](#_Toc29597)

[Figure S25. TEM images of TTED-Pt-AF.	38](#_Toc22520)

[.	38](#_Toc32000)

[Figure S26. (a) HAADF-STEM image of Pt centers in TTED-Pt-AF and (b) corresponding EDS elemental mapping of Pt.	39](#_Toc18576)

[Figure S27. TG curves of TTED-M-AFs and TTED-GDY.	40](#_Toc25364)

[Figure S28. (a) KPFM surface potential image of TTED-GDY. The piezoelectric response amplitude-voltage (b) butterfly loops and (c) hysteresis loops—phase-voltage demonstrate reversible and switchable domain behavior for TTED-GDY.	41](#_Toc21597)

[Figure S29. (a) Transient piezoelectric current response of TTED-M-AFs under periodic mechanical stimulation. (b) EIS profiles, (c) PL spectra, and LSV curves under (d) Ar and (e) CO](#_Toc21682)_[2](#_Toc21682)_ [atmospheres for TTED-GDY and TTED-M-AFs.	42](#_Toc21682)

[Figure S30. Bandgap energies and corresponding DRS spectra (inset) of TTED-GDY.	43](#_Toc21272)

[Figure S31. Photographic images of TTED-M-AFs and TTED-GDY samples.	44](#_Toc9702)

[Figure S32. M−S plots of (a) TTED-Pt-AF, (b) TTED-Pd-AF, and (c) TTED-Ni-AF recorded at various frequencies. VB-XPS spectra of (d) TTED-Pt-AF, (e) TTED-Pd-AF, and (f) TTED-Ni-AF.	45](#_Toc7915)

[Figure S33. Piezoelectric CO](#_Toc3390)_[2](#_Toc3390)_ [reduction yield of TTED-Pt-AF (a) as a function of varying Na](#_Toc3390)_[2](#_Toc3390)_[SO](#_Toc3390)_[3](#_Toc3390)_ [sacrificial agent dosage, and (b) with different catalyst loadings.	46](#_Toc3390)

[Figure S34.](#_Toc27921) ^[1](#_Toc27921)^[H NMR spectrum of reacted liquid.	47](#_Toc27921)

[Figure S35. PL spectra of TTED-Pt-AF compared to TTED-Pt-AF in the presence of Na](#_Toc26721)_[2](#_Toc26721)_[SO](#_Toc26721)_[3](#_Toc26721)_[.	48](#_Toc26721)

[Figure S36. FTIR spectra of fresh and reacted TTED-Pt-AF samples.	49](#_Toc5255)

[Figure S37. (a) Survey XPS spectra of TTED-Pt-AF before and after catalysis. High-resolution core-level XPS spectra of reacted TTED-Pt-AF for (b) C 1s,](#_Toc10885) [(c) P](#_Toc10885) [2p,](#_Toc10885) [and (d) Pt 4f regions.	50](#_Toc10885)

[Figure S38. SEM images and EDS elemental mapping of reacted TTED-Pt-AF.	51](#_Toc31532)

[Figure S39. PXRD patterns of fresh and reacted TTED-Pt-AF samples.	52](#_Toc27523)

[Figure S40. (a) In situ high-pressure FTIR spectrum of TTED-GDY, capturing molecular vibrational changes under applied pressure. (b) Deconvolution and fitting of corresponding infrared peaks of TTED-GDY.	53](#_Toc25862)

[Figure S41. Microphotographs of TTED-Pt-AF recorded under selected pressures during irradiation with a 355 nm laser.	54](#_Toc1176)

[Figure S42. Microphotographs of TTED-Pd-AF captured at various applied pressures under irradiation with a 355 nm laser.	55](#_Toc32387)

[Figure S43. Microphotographs of TTED-Ni-AF recorded at selected pressures under irradiation with a 355 nm laser.	56](#_Toc20495)

[Figure S44. Microphotographs of TTED-GDY recorded at selected pressures under irradiation with a 355 nm laser.	57](#_Toc496)

[Figure S45. (a) In situ high-pressure PL spectra of TTED-GDY. (b) Variation of PL peak position (pink dots) and intensity (green dots) as a function of pressure for TTED-GDY.	58](#_Toc2927)

[Figure S46. Optimized geometric structures and corresponding chemical adsorption energies of CO](#_Toc6525)_[2](#_Toc6525)_ [on (a) TTED organic center and (b) the −C≡C−M(PEt](#_Toc6525)_[3](#_Toc6525)_[)](#_Toc6525)_[2](#_Toc6525)_[−C≡C− moiety within TTED-M-AFs under an applied pressure of 2 GPa.	59](#_Toc6525)

[Figure S47. The ESP surface maps for optimized structures of TTED-M-AFs.	60](#_Toc6112)

[Figure S48. Optimized molecular structures depicting the catalytic reduction of CO](#_Toc27267)_[2](#_Toc27267)_ [to CO at the −C≡C−M(PEt](#_Toc27267)_[3](#_Toc27267)_[)](#_Toc27267)_[2](#_Toc27267)_[−C≡C− active sites within TTED-M-AFs under ambient pressure.	61](#_Toc27267)

[Figure S49. Optimized molecular structures depicting the catalytic reduction of CO](#_Toc29407)_[2](#_Toc29407)_ [to CO at the −C≡C−M(PEt](#_Toc29407)_[3](#_Toc29407)_[)](#_Toc29407)_[2](#_Toc29407)_[−C≡C− active sites within TTED-M-AFs under 2 GPa.	62](#_Toc29407)

[Figure S50. Measured H](#_Toc23537)_[2](#_Toc23537)_ [production rates of TTED-M-AFs under piezocatalytic conditions.	63](#_Toc23537)

[Table S1. EXAFS fitting parameters at the Pt](#_Toc20862) *[L](#_Toc20862)_[3](#_Toc20862)_*[-edge for various samples.	64](#_Toc20862)

[Table S2 Measured metal content of TTED-M-AFs determined by ICP-MS, compared with calculated theoretical values.	65](#_Toc28610)

[Table S3 A comparative analysis of piezoelectric coefficients (](#_Toc32448)*[d](#_Toc32448)_[33](#_Toc32448)_*[) between TTED-M-AFs in our work and previously reported 2D materials.	66](#_Toc32448)

[Table S4 Calculated LUMO and HOMO energy levels of TTED-M-AFs under ambient conditions and upon application of 2 GPa mechanical pressure.	67](#_Toc29241)

[4. References	68](#_Toc15012)

# 1 General Information

## 1.1 Chemicals

All reagents used in the reactions were obtained from commercial suppliers and used as received without further purification or modification. 2,7,10,15-Tetrakis((trimethylsilyl)ethynyl)dibenzo[g,p]chrysene (TTED-TMS) was purchased from Yanshen (Extension) Technology Corporation Limited. Copper(I) iodine (CuI, ACS reagent, 98%) was purchased from J&K. Triethylphosphine (PEt_3_) in tetrahydrofuran (THF, 1 M solution), diethyl sulfide (SEt_2_), and tetrabutylammonium fluoride (TBAF, 1.0 M in THF) were obtained from Sigma Aldrich. THF (99.5%, extra dry), triethylamine (Et_3_N, 99.5%, extra dry), and dimethyl sulfoxide (DMSO, 99.7%, extra dry) were sourced from Energy Chemical. Absolute ethanol (HPLC grade, ≥ 99.8%) was sourced from Macklin. Platinum chloride (PtCl_2_), palladium chloride (PdCl_2_), sodium chloride (NaCl), sodium sulfite (Na_2_SO_3_), and potassium bromide (KBr) were provided by Deckman. Nickel(II) chloride hexahydrate (NiCl_2_⋅6H_2_O, 98+%) was supplied by ACROS. Dichloromethane (DCM, ACS grade), chloroform (CH_2_Cl_2_, ACS grade), methanol (CH_3_OH, ACS grade), acetonitrile (CH_3_CN, ACS grade), and THF (ACS grade) were purchased from Damas-beta. Deuterated chloroform (CDCl_3_, 99.8%) was provided by Cambridge Isotope Laboratories, Inc. silica gel (200-mesh) was purchased from Huanghai Brand.

## 1.2 Characterization techniques

Scanning electron microscopy (SEM) and energy dispersive spectroscopy (EDS) mapping were performed using a Tescan MIRA microscope. Transmission electron microscopy (TEM) and high-resolution TEM (HRTEM) analyses were conducted with a JEOL JEM-2100F instrument. Powder X-ray diffraction (PXRD) patterns were conducted using a SmartLab 9 kW Rigaku diffractometer over a 2θ range of 2°–40° at a scan rate of 2° min^−1^. X-ray photoelectron spectroscopy (XPS) measurements employed a Thermo Escalab 250XI system with K-Alpha radiation (12 kV, 6 mA filament current) under ultrahigh vacuum (2.0 × 10^−7^ mbar), with spectra calibrated to the C 1s peak at 284.4 eV. Fourier transform infrared (FTIR) spectra were collected using a Thermo Scientific Nicolet IS50 FTIR Advanced XT-KBR Gold Spectrometer. Nuclear magnetic resonance (NMR) spectroscopy including ^1^H, ^13^C, and ^31^P NMR were recorded on a Bruker AVANCE-III 600 MHz spectrometer, while solid-state ^13^C cross-polarization magic angle spinning (CP-MAS) NMR was performed on a Jeol ECZ500R 500 MHz solid-state NMR instrument. Piezoelectric force microscopy (PFM) and Kelvin probe force microscopy (KPFM) were conducted using a Bruker Multimode 8 system. Electrochemical measurements were performed on a CHI660E electrochemical workstation. In situ diffuse reflectance infrared Fourier transform spectroscopy (DRIFTS) was conducted using a Thermo Fisher CRCP-7070-I spectrometer. Specific surface areas were determined by nitrogen adsorption using an ASAP2460 surface area and porosimetry analyzer. Ultraviolet-visible diffuse reflectance spectroscopy (UV-Vis DRS) was acquired with a Shimadzu UV-2600 spectrometer. Thermogravimetric analysis (TGA) was conducted on a Mettler Toledo TGA/DSC3+ instrument. In situ high-pressure PL and IR spectroscopy utilized a custom-built system combining an optical fiber spectrometer with a Nicolet iN10 microscope spectrometer (Thermo Fisher Scientific, USA). These in situ experiments were performed in collaboration with Light & Microvision Industrial Technology Co., Ltd. The PL spectra of TTED-M-AFs in aqueous suspension was recorded under excitation at 420 nm using an Agilent G9800A Cary Eclipse spectrofluorometer. The piezo-catalytic experiments were conducted using a 100 W ultrasound generator (Shumei, KQ-100)_._ The concentrations of CO and H_2_ were quantified using a gas chromatograph (SHIMADZU GC-2030). Isotope labeling experiments employing ^13^C were analyzed by gas chromatography-mass spectrometry (GC MS QP-2020 NX, SHIMADZU).

# 2 Experimental Sections

## 2.1 Synthesis of *trans*-Ni(PEt_3_)_2_Cl_2_

The *trans*-Ni(PEt_3_)_2_Cl_2_ complex can be synthesized according to the literature method [1, 2]. Specifically, in a nitrogen-protected two-neck round-bottom flask (RBF) equipped with a magnetic stirrer, 0.4747 g (3.7 mmol) of NiCl_2_·6H_2_O was dissolved in 8 mL of EtOH. N_2_ gas was bubbled through the solution for 30 min to remove dissolved oxygen. Subsequently, 7.4 mL (7.4 mmol) of a 1 M PEt_3_ solution in THF was added dropwise, during which the solution color changed from bright green to deep red. The reaction mixture was heated to 80 ℃ and stirred under N_2_ for 1 h. After naturally cooling to room temperature, an ice bath was applied with continuous stirring for 1 h to facilitate recrystallization. The resulting *trans*-Ni(PEt_3_)_2_Cl_2_ was isolated by vacuum suction filtration using a 0.22 μm membrane filter, yielding 72.3 wt% of the product. ^1^H NMR (600 MHz, Chloroform-*d*) δ: 1.68 (q, *J* = 7.6 Hz, 1H), 1.32 (t, *J* = 7.5 Hz, 2H). ^13^C NMR (151 MHz, Chloroform-*d*) δ: 13.25, 8.25. ^31^P NMR (243 MHz, Chloroform-*d*) δ: 5.47.

## 2.2 Synthesis of *trans*-Pd(PEt_3_)_2_Cl_2_

The *trans*-Pd(PEt_3_)_2_Cl_2_ complex was prepared from PdCl_2_, NaCl, and PEt_3_ as depicted in **Scheme S1**. PdCl_2_ (0.355 g, 2.0 mmol) and NaCl (0.240 g, 4.0 mmol) were combined in a two-neck RBF with 8 mL of deionized water and the mixture was stirred at 100 °C for 1 hour. Upon cooling to room temperature (RT), 4.1–4.3 mL (4.1–4.3 mmol) of PEt_3_ solution was added dropwise over 2 hours. The solution gradually changed from purple to light yellow. The reaction mixture was concentrated by rotary evaporation, and the solid *trans*-Pd(PEt_3_)_2_Cl_2_ was collected via vacuum suction filtration on a 0.22 μm filter, and then washed with water. The complex was obtained in 82.4 wt% yield. The synthetic process of *trans*-Pd(PEt_3_)_2_Cl_2_ was presented in **Scheme S1**. Characterization by ^1^H, ^13^C, and ^31^P NMR is provided in **Figure S1**. ^1^H NMR (600 MHz, Chloroform-*d*) δ: 1.87 (pd, *J* = 7.3, 4.5 Hz, 1H), 1.19 (p, *J* = 8.1 Hz, 2H). ^13^C NMR (151 MHz, Chloroform-*d*) δ: 13.78 (t, *J* = 13.7 Hz), 8.06. ^31^P NMR (243 MHz, Chloroform-*d*) δ: 17.84. ESI-MS calcd for [M−Cl]^+^: 378.17, found 379.05.

**Scheme** **S1.** Synthetic process of *trans*-Pd(PEt_3_)_2_Cl_2_.

## 2.3 Synthesis of *trans*-Pt(PEt_3_)_2_Cl_2_

The *trans*-Pt(PEt_3_)_2_Cl_2_ could be synthesized according to the literature method [3]. Specifically, PtCl_2_ (0.532 g, 2.0 mmol) was placed in a nitrogen-purged two-neck RBF containing 20 mL of CH_2_Cl_2_ and 2.2 mL (20 mmol) SEt_2_, and the mixture was stirred at room temperature for 30 minutes. Subsequently, 4.1–4.3 mL (4.1–4.3 mmol) of PEt_3_ was added dropwise at room temperature over 2 hours. After rotary evaporation, the crude solid was purified via column chromatography on 200-mesh silica gel. The obtained *cis*-Pt(PEt_3_)_2_Cl_2_ was then converted to *trans*-Pt(PEt_3_)_2_Cl_2_ by heating at 185 °C for 5 hours. The final yield was 70.2% of the product. ^1^H NMR (600 MHz, Chloroform-*d*) δ: 1.90 (dt, *J* = 12.8, 5.6 Hz, 1H), 1.18 (p, *J* = 8.0 Hz, 2H). ^13^C NMR (151 MHz, Chloroform-*d*) δ: 12.70 (t, *J* = 16.7 Hz), 7.70. ^31^P NMR (243 MHz, Chloroform-*d*) δ: 17.15, 12.22, 7.29. ESI-MS calcd for [M−2Cl+H]^+^: 432.39, found 433.15.

## 2.4 Synthesis of TTED-M-AFs

The synthetic route for TTED-M-GYs is illustrated in **Scheme S2**. The trimethylsilyl protecting groups of 2,7,10,15-tetrakis((trimethylsilyl)ethynyl)dibenzo[g,p]chrysene (TTED-TMS) were removed using TBAF solution to yield 2,7,10,15-tetrakis(ethynyl)dibenzo[g,p]chrysene (TTED) (**Figure S2**). ^1^H NMR (600 MHz, Chloroform-*d*) δ: 3.76–3.70 (m, 2H), 2.18 (s, 1H), 1.25 (t, *J* = 7.0 Hz, 4H). ^13^C NMR (151 MHz, Chloroform-*d*) δ: 58.51, 18.45. MALDI-TOF [M+H]^+^: 425.13, found 424.96.

For TTED-M-AFs synthesis, 0.365 g (1.0 mmol) *trans*-Ni(PEt_3_)_2_Cl_2_, 0.502 g (1.0 mmol) *trans*-Pt(PEt_3_)_2_Cl_2_, or 0.412 g (1.0 mmol) *trans*-Pd(PEt_3_)_2_Cl_2_ was separately mixed with 0.212 g (0.5 mmol) TTED and 0.6 mg (0.003 mmol) CuI, followed by addition of 5 mL Et_3_N and 20 mL THF in a 50 mL Schlenk tube equipped with a magnetic stirrer. After three freeze-pump-thaw cycles to remove O_2_, the mixtures were heated sequentially at 30 ℃ for 24 hours, 60 ℃ for 48 hours, and 80 ℃ for 24 hours. The resulting solids were isolated by vacuum filtration and purified by Soxhlet extraction with THF, CH_3_OH, and CH_3_CN for 24 hours each to remove residual Ni^II^-salt, CuI, unreacted monomers, and oligomers. The isolated yields of TTED-Ni-AF, TTED-Pt-AF, and TTED-Pd-AF were 81.3 wt%, 83.6 wt% and 80.5 wt%, respectively.

**Scheme S2.** Synthetic route of TTED-M-AFs.

## 2.5 Synthesis of TTED-GDY

To assess the influence of the M^II^-(PEt_3_)_2_ units in TTED-M-AFs, a control sample TTED-GDY was synthesized following the route in **Scheme S3**. A mixture of 424 mg (1.0 mmol) TTED, 0.13 mg (0.06 mmol) CuI, and 10 mL DMSO was sealed in a Schlenk tube and heated at 80 ℃ for 3 days. Post-synthesis purification was conducted by Soxhlet extraction with CH_3_CN and THF for 24 hours each to remove the CuI, unreacted monomers and oligomers, respectively. TTED-GDY was obtained in 78.5 wt% yield.

**Scheme S3.** Synthetic route of TTED-GDY.

## 2.6 In situ high-pressure PL and IR absorption spectroscopies

High-pressure measurements were implemented using a symmetric diamond anvil cell (DAC) equipped with ultra-low fluorescence diamonds featuring 400 µm culet diameters. Samples and a ruby pressure calibrant sphere were loaded into a 150 µm diameter cavity laser-drilled in a T301 stainless steel gasket pre-indented to 45 µm thickness. Pressure calibration was achieved via the ruby fluorescence method by monitoring the shift of the R1 fluorescence line. Silicone oil was employed as the pressure-transmitting medium to ensure hydrostatic conditions. All experiments were conducted at ambient temperature.

## 2.7 Preparation of nanosheets for AFM characterization

The Pt^II^-acetylide framework (TTED-Pt-AF) (1 mg) was dispersed in ethanol (5 mL) and subjected to bath sonication (200 W) for 48 h while maintained in an ice bath to prevent thermal degradation. To isolate monolayer and few-layer nanosheets, the resulting suspension was centrifuged at 6000 rpm for 5 min to remove unexfoliated bulks. The supernatant containing the exfoliated nanosheets was carefully collected for subsequent AFM analysis.

## 2.8 PFM and KPFM measurements

Powdered samples were dispersed in ethanol via ultrasonication for 5 minutes. A 60 μL aliquot of the suspension was drop-cast onto a 1 × 1 cm^2^ indium tin oxide (ITO) substrate, which was electrically connected to a round iron plate using conductive silver paste. Piezoelectric amplitude and phase mappings of TTED-M-AFs were obtained using a Bruker Multimode 8 atomic force microscope (AFM) operating in PFM mode. KPFM measurements were conducted using AFM in electrochemical mode with Pt/Ir-coated tips. The piezoelectric coefficient (*d*₃₃) was calculated according to Equation (1) [4]:

$\text{d}_{\text{33}}\text{=}\frac{\text{A}_{\text{1}}\text{-}\text{A}_{\text{2}}}{\text{16×}\text{(}\text{V}_{\text{1}}\text{-}\text{V}_{\text{2}}\text{)}}\text{}\text{ }$	(1)

where *A_1_* and *V_1_* denote the amplitude and voltage values at the intersection of the hysteresis loop, respectively. *A_2_* and *V_2_* denote the respective values at different points of the hysteresis loop, respectively.

## 2.9 Carrier transfer behavior assessment

The carrier transfer characteristics of TTED-M-AFs were evaluated by combining piezoelectric current measurements with linear sweep voltammetry (LSV) in a neutral electrolyte. In a typical procedure, 10 mg of catalyst was dispersed in 40 mL of 0.5 M Na_2_SO_4_. The suspension was magnetically stirred to ensure homogeneous dispersion, and the stirring speed was varied to probe shear- and contact-induced effects on charge transport. During activation of stirring, the generated piezoelectric current was recorded in real time, and stepwise LSV measurements were performed to correlate electrochemical response with the observed piezocurrent. For the preparation of samples for Mott-Schottky (M−S) test, a specific procedure was followed: 1.0 mg catalyst was dispersed in a mixture containing 0.75 mL of H_2_O, 0.25 mL of isopropyl alcohol and 0.03 mL of Nafion, then followed by ultrasonic treatment for 10 min. Subsequently, the suspension was carefully dropped onto an ITO plate and dried in an oven to form a film measuring 1 × 1 cm^2^. The piezoelectric current, LSV test and M−S test were conducted using a CHI660E electrochemical workstation. The setup for these tests included the as-synthesized samples serving as the working electrode, platinum wires as the counter electrode, and an Ag/AgCl calomel electrode as the reference electrode, with the electrolyte being the medium for the reactions.

## 2.10 Calculation of energy bands for TTED-M-AFs

The conduction bands (CBs) for TTED-M-AFs were calculated by the equation below:

$\text{E}_{\text{NHE}\left( \text{V} \right)}\text{ = }\text{E}_{\text{Ag}\text{/}\text{AgCl}}\text{+0.24}$	(2)

$\text{E}_{\text{Ag}\text{/}\text{AgCl}}$ are −0.52 V, −0.51 V and −0.47 V for TTED-Pt-AF, TTED-Pd-AF and TTED-Ni-AF, respectively. Therefore, the flat band energies $\text{E}_{\text{NHE}\left( \text{V} \right)}$for TTED-Pt-AF, TTED-Pd-AF, and TTED-Ni-AF are −0.28 V, −0.27 V and −0.23 V, respectively. Valence band (VB) energy for TTED-Pt-AF, TTED-Pd-AF, and TTED-Ni-AF are obtained from VB-XPS, being 1.40 eV, 1.15 eV, and 1.10 eV, respectively. Furthermore, the band gap energy is 2.12 eV, 2.0 eV and 1.85 eV, so the conduction band (CB) energy can be calculated to be −0.72 eV, −0.90 eV and −0.70 eV for TTED-Pt-AF, TTED-Pd-AF, and TTED-Ni-AF, respectively.

## 2.11 Calculation of CO production and selectivity

The calculation of CO production was shown below,

$\text{V}_{\text{CO}}\text{= }\frac{\text{A}_{\text{CO}}}{\text{7103876.45361×0.3 }}\text{×17}$	(3)

$\text{m}_{\text{CO}}\text{=}\frac{\text{V}_{\text{CO}}}{\text{22.4}}\text{×1000}$	(4)

where $\text{A}_{\text{CO}}$ represents CO peak integral areas in gas chromatography, respectively. $\text{V}_{\text{CO}}$ is CO volume in reaction bottle, respectively. $\text{m}_{\text{CO}}$ is CO yield (μmol), respectively. The selectivity (*S*) of products was calculated by the following equations:

$\text{ }\text{S}_{\text{CO}}\text{=}\frac{\text{m}_{\text{CO}}}{\text{m}_{\text{co}}\text{+}\text{m}_{\text{H}_{\text{2}}}}$	(5)

## 2.12 DFT simulations

The TTED-M-AFs structural model was constructed using the Materials Studio software suite. Initially, the lattice was based on the *P*1 space group, with *a* and *b* lattice parameters set at 29.6744 Å for TTED-Pt-AF, 31.4675 Å for TTED-Pd-AF, and 31.5480 Å for TTED-Ni-AF. These values were determined by measuring the center-to-center distances between the vertices of TTED-M-AFs. The structure was then optimized geometrically using the Forcite module, which utilizes Universal force fields and Ewald summations to ensure precise structural refinement. After optimization, the Reflex Plus module was used to calculate the Powder X-Ray Diffraction (PXRD) pattern. Finally, the Pawley refinement technique was applied to fine-tune the PXRD profile, yielding the refined PXRD pattern along with the *R_wp_* and *R_p_* values, which serve as indicators of the fit quality.

First-principles calculations were performed using the Vienna ab initio Simulation Package (VASP) within the generalized gradient approximation (GGA) employing the Perdew-Burke-Ernzerhof (PBE) functional [5]. The projector augmented wave (PAW) method described electron-ion interactions. A kinetic energy cutoff of 450 eV was used for all calculations. Structural models of TTED-Ni-AF, TTED-Pd-AF, and TTED-Pt-AF were constructed with lattice parameters *a* = *b* = *c* = 10.00 Å, incorporating a 15 Å vacuum layer along the vertical direction to avoid interlayer interactions. Initial pressure conditions were referenced from prior studies and set to 2 GPa using Materials Studio for preliminary optimization [6]. Structures were then further optimized in VASP with an energy convergence criterion of 10^−5^ eV and atomic forces restrained below 0.05 eV Å^−1^ [7]. A Hubbard U correction of 4 eV was applied to Ni 3*d* states to more accurately capture electron correlation effects.

Adsorption energy (*ΔE_ads_*) were calculated by：

*ΔE_ads_ = E_ad/sub_ – E_ad_* *– E_sub_*	(6)

where *E_ad/sub_*, *E_ad_*, and *E_sub_* correspond to the total energy of the adsorbate-substrate system, isolated adsorbate, and clean substrate, respectively. More negative *ΔE_ads_* values indicate stronger adsorption.

Free energy changes (*ΔG*) were computed according to:

*ΔG = ΔE + ΔZPE –TΔS*	(7)

Where *ΔE* is the DFT total energy, *ΔZPE* the zero-point energy correction, *T* the temperature (300 K), and Δ*S* the entropy change.

The chosen pressure of 2 GPa corresponds to the cavitation-induced pressures generated during ultrasonic irradiation. Ultrasonication produces cyclic compression and decompression phases in aqueous media leading to bubble formation and collapse, which can generate localized pressures up to 0.1–2 GPa. Prior studies and theoretical models confirm that under typical ultrasonic conditions (e.g., 40 kHz, 100 W), comparable pressures are achievable, justifying the selection of 2 GPa for pressure-dependent simulations.

In a cavitation bubble, the total pressure *P* can be estimated by:

$\text{P}\text{=}\text{P}_{\text{0}}\text{ + }{\text{2}\text{σ}\text{/}\text{R}}_{\text{0}}$	(8)

where $\text{P}_{\text{0}}$ is the ambient pressure, σ the surface tension, and $\text{ }\text{R}_{\text{0}}$ the initial bubble radius.

Experimental evidence shows that at temperatures of 298 K and ambient pressure of 1 atm, cavitation bubbles with radii around 50 μm can generate pressures near 2 GPa at ultrasound frequencies around 19.5 kHz, supporting the relevance of the simulation conditions used here [8].

# 3 Supplementary Figures and Tables

## Figure S1. (a) ^1^H, (b) ^13^C and (c) ^31^P NMR spectra of *trans*-Pd(PEt_3_)_2_Cl_2_.

Figure S2. (a) ^1^H and (b) ^13^C NMR spectra of TTED ligand.

## Figure S3. Simulated PXRD patterns of TTED-Pt-AF for AA and AB stacking configurations.

## Figure S4. (a) Simulated PXRD patterns of TTED-Pd-AF for AA and AB stacking configurations. (b) Experimental PXRD pattern of TTED-Pd-AF (red) overlaid with the profile calculated from Pawley refinement (blue), along with the difference curve (residual, yellow), compared to the simulated pattern derived from the optimized structural model (green). Reflection positions are indicated by vertical tick marks. (c) Approximate unit cell parameters from Pawley refinement of TTED-Pd-AF.

## Figure S5. (a) Simulated PXRD patterns of TTED-Ni-AF for AA and AB stacking configurations. (b) Experimental PXRD pattern of TTED-Ni-AF (red), Pawly refinement simulated profile (blue), residual difference curve (yellow), and the simulated pattern from the optimized structural model (green). Reflection positions are denoted by vertical tick marks. (c) Approximate unit cell parameters from Pawley refinement of TTED-Ni-AF.

## Figure S6. HR-TEM images of TTED-Pd-AF: (a) Lattice fringes corresponding to the (1 0 2) plane and (b) interlayer spacing resolved within the stacked framework.

## Figure S7. HR-TEM images of TTED-Ni-AF: (a) Lattice fringes corresponding to the (0 0 2) plane and (b) interlayer spacing resolved within the stacked framework.

## Figure S8. HR-TEM images of TTED-Pt-AF. (a) Lateral view highlighting the crystallographic plane (2 0 1), (b) and (c) magnified HR-TEM images of TTED-Pt-AF, showing local short-range order and local pore‑like features.

## Figure S9. Raman spectra of TTED-M-AFs.

## Figure S10. Solid-state ^13^C CP-MAS NMR spectra of (a) TTED-Pd-AF and (b) TTED-Ni-AF.

## Figure S11. Solid-state ^31^P NMR spectrum of TTED-Pt-AF.

## Figure S12. N_2_ adsorption-desorption isotherms along with corresponding pore size distributions for (a) TTED-Pt-AF, (b) TTED-Pd-AF, and (c) TTED-Ni-AF. The isotherms display characteristic physisorption behavior, and the derived pore size distributions reveal narrow microporous channels centered around 0.78 nm across all samples.

## Figure S13. Bulk powder volume of TTED-M-AFs (M = Pt, Pd, Ni), illustrating the characteristic low-density morphology of layered frameworks.

## Figure S14. Calculated average pore diameter distribution for TTED-Pt-AF based on structural model.

## Figure S15. Calculated average pore diameter distribution for TTED-Pd-AF based on the structural model.

## Figure S16. Calculated average pore diameter distribution for TTED-Ni-AF based on the structural model.

Figure S17. (a) Survey XPS spectra of TTED-M-AFs (M = Pt, Pd, Ni). (b) High-resolution P 2p core-level XPS spectrum of TTED-Pt-AF. (c) High-resolution Pt 4f core-level XPS spectrum of TTED-Pt-AF. (d) High-resolution C 1s core-level XPS spectrum of TTED-Ni-AF. (e) P 2p core-level XPS spectrum of TTED-Ni-AF. (f) Ni 2p core-level XPS spectrum of TTED-Ni-AF. (g) C 1s core-level XPS spectrum of TTED-Pd-AF. (h) P 2p core-level XPS spectrum of TTED-Pd-AF. (i) Pd 3d core-level XPS spectrum of TTED-Pd-AF.

## Figure S18. (a) Survey XPS spectra of TTED-GDY. (b) High-resolution C 1s core-level XPS spectrum of TTED-GDY.

## Figure S19. Linear fitting of Pt white-line intensity for standard samples, establishing a calibration curve used to determine the oxidation state of Pt in TTED-Pt-AF.

## Figure S20. EXAFS analysis at the Pt *L_3_*-edge for TTED-Pt-AF and reference materials. Fourier-transformed EXAFS spectra (*R*-space, magnitude) with experimental data (points) and theoretical fits (lines) for (a) TTED-Pt-AF, (b) PtO_2_, and (c) Pt foil. (d) *k^3^*-weighting EXAFS spectra (*k*-space) of Pt foil, PtO_2_, and TTED-Pt-AF. Corresponding *k*-space fitting curves (lines) overlaid on experimental data (points) for (e) TTED-Pt-AF, (f) PtO_2_, and (g) Pt foil. 3D wavelet transform plots of the *k^3^*-weighted Pt *L_3_*-edge EXAFS spectra for (h) Pt foil and (i) PtO_2_, respectively.

## Figure S21. SEM images of TTED-Pt-AF, accompanied by EDS elemental mapping for C, Pt, and P.

## Figure S22. SEM images of TTED-Pd-AF, accompanied by EDS elemental mapping for C, Pd, and P.

## Figure S23. SEM images of TTED-Ni-AF, accompanied by EDS elemental mapping for C, Ni, and P.

## Figure S24. SEM images of TTED-GDY, accompanied by EDS elemental mapping for C.

## Figure S25. TEM images of TTED-Pt-AF.

## .

## Figure S26. (a) HAADF-STEM image of Pt centers in TTED-Pt-AF and (b) corresponding EDS elemental mapping of Pt.

## Figure S27. TG curves of TTED-M-AFs and TTED-GDY.

## Figure S28. (a) KPFM surface potential image of TTED-GDY. The piezoelectric response amplitude-voltage (b) butterfly loops and (c) hysteresis loops—phase-voltage demonstrate reversible and switchable domain behavior for TTED-GDY.

## Figure S29. (a) Transient piezoelectric current response of TTED-M-AFs under periodic mechanical stimulation. (b) EIS profiles, (c) PL spectra, and LSV curves under (d) Ar and (e) CO_2_ atmospheres for TTED-GDY and TTED-M-AFs.

## Figure S30. Bandgap energies and corresponding DRS spectra (inset) of TTED-GDY.

## Figure S31. Photographic images of TTED-M-AFs and TTED-GDY samples.

## Figure S32. M−S plots of (a) TTED-Pt-AF, (b) TTED-Pd-AF, and (c) TTED-Ni-AF recorded at various frequencies. VB-XPS spectra of (d) TTED-Pt-AF, (e) TTED-Pd-AF, and (f) TTED-Ni-AF.

## Figure S33. Piezoelectric CO_2_ reduction yield of TTED-Pt-AF (a) as a function of varying Na_2_SO_3_ sacrificial agent dosage, and (b) with different catalyst loadings.

## Figure S34. ^1^H NMR spectrum of reacted liquid.

## Figure S35. PL spectra of TTED-Pt-AF compared to TTED-Pt-AF in the presence of Na_2_SO_3_.

## Figure S36. FTIR spectra of fresh and reacted TTED-Pt-AF samples.

## Figure S37. (a) Survey XPS spectra of TTED-Pt-AF before and after catalysis. High-resolution core-level XPS spectra of reacted TTED-Pt-AF for (b) C 1s, (c) P 2p, and (d) Pt 4f regions.

## Figure S38. SEM images and EDS elemental mapping of reacted TTED-Pt-AF.

## Figure S39. PXRD patterns of fresh and reacted TTED-Pt-AF samples.

## Figure S40. (a) In situ high-pressure FTIR spectrum of TTED-GDY, capturing molecular vibrational changes under applied pressure. (b) Deconvolution and fitting of corresponding infrared peaks of TTED-GDY.

## Figure S41. Microphotographs of TTED-Pt-AF recorded under selected pressures during irradiation with a 355 nm laser.

## Figure S42. Microphotographs of TTED-Pd-AF captured at various applied pressures under irradiation with a 355 nm laser.

## Figure S43. Microphotographs of TTED-Ni-AF recorded at selected pressures under irradiation with a 355 nm laser.

## Figure S44. Microphotographs of TTED-GDY recorded at selected pressures under irradiation with a 355 nm laser.

## Figure S45. (a) In situ high-pressure PL spectra of TTED-GDY. (b) Variation of PL peak position (pink dots) and intensity (green dots) as a function of pressure for TTED-GDY.

## Figure S46. Optimized geometric structures and corresponding chemical adsorption energies of CO_2_ on (a) TTED organic center and (b) the −C≡C−M(PEt_3_)_2_−C≡C− moiety within TTED-M-AFs under an applied pressure of 2 GPa.

## Figure S47. The ESP surface maps for optimized structures of TTED-M-AFs.

## Figure S48. Optimized molecular structures depicting the catalytic reduction of CO_2_ to CO at the −C≡C−M(PEt_3_)_2_−C≡C− active sites within TTED-M-AFs under ambient pressure.

## Figure S49. Optimized molecular structures depicting the catalytic reduction of CO_2_ to CO at the −C≡C−M(PEt_3_)_2_−C≡C− active sites within TTED-M-AFs under 2 GPa.

## Figure S50. Measured H_2_ production rates of TTED-M-AFs under piezocatalytic conditions.

## Table S1. EXAFS fitting parameters at the Pt *L_3_*-edge for various samples.

| **Sample** | **Shell** | **CN^a^** | **R (Å)^b^** | σ^2^ (Å^2^)^c^ | ΔE_o_ (eV)^d^ | K-range (Å) | R-range (Å) | R factor |
| --- | --- | --- | --- | --- | --- | --- | --- | --- |
| Pt foil | Pt−Pt | 12* | 2.76±0.01 | 0.0043±0.0002 | 7.5±0.2 | 3.0−13.0 | 1.2−3.2 | 0.0024 |
| PtO_2_ | Pt−O | 6* | 1.99±0.01 | 0.0029±0.0017 | 8.9±0.7 | 3.0−10.5 | 1.2−2.2 | 0.0084 |
| TTED-Pt-AF | Pt−C | 2.0±0.4 | 1.96±0.01 | 0.0056±0.0020 | 5.2±0.7 | 3.0−12.0 | 1.2−2.4 | 0.0090 |
|  | Pt−P | 2.9±0.5 | 2.30±0.01 |  |  |  |  |  |

CN^a^: Average coordination number; R^b^: Interatomic distance between absorbing (central) atom and backscattering atoms; σ^2c^: Debye-Waller factor to account for both thermal and structural disorders; ΔE_o_^d^: Energy shift correction accounting for inner potential misalignment between theoretical phase functions and experimental absorption edge.

## Table S2 Measured metal content of TTED-M-AFs determined by ICP-MS, compared with calculated theoretical values.

| **TTED-M-AFs** | **Metal** | **Experimental value (wt%)** | **Calculated value (wt%)** |
| --- | --- | --- | --- |
| TTED-Pt-AF | Pt | 23.63 | 27.54 |
| TTED-Pd-AF | Pd | 12.29 | 15.89 |
| TTED-Ni-AF | Ni | 8.50 | 10.67 |

## Table S3 A comparative analysis of piezoelectric coefficients (*d_33_*) between TTED-M-AFs in our work and previously reported 2D materials.

| **2D crystal** | **Piezoelectric coefficient *d_33_* (pm V^−1^)** | **Reference** |
| --- | --- | --- |
| TTED-Pt-AF | 35.0 | **This work** |
| TTED-Pd-AF | 21.9 | **This work** |
| TTED-Ni-AF | 9.25 | **This work** |
| HETP-Ni-GY | 4.91 | [2] |
| HETP-GDY | 4.28 | [2] |
| MXene | 0.78 | [9] |
| BiOIO_3_ | 1.72 | [10] |
| g-C_3_N_4_ | 2.29 | [10] |
| Pd_4_S_3_Se_3_ | 3.92 | [11] |
| Ni_4_S_3_Se_3_ | 2.53 | [11] |
| GaN | 3.1 | [12] |
| MoS_2_ flake | 0.9 | [13] |
| In_2_Se_3_ | 9.19 | [14] |
| SnS_2_ | 5.0 | [15] |
| CdS | 12.0 | [16] |
| CityU-13 | 20.9 | [17] |
| CityU-14 | 18.9 | [17] |
| NH_2_-MIL-125 | 11.75 | [18] |
| Cu-NH_2_-MIL-125 | 26.21 | [18] |

## Table S4 Calculated LUMO and HOMO energy levels of TTED-M-AFs under ambient conditions and upon application of 2 GPa mechanical pressure.

|  | **TTED-Ni-AF** | **TTED-Pd-AF** | **TTED-Pt-AF** |
| --- | --- | --- | --- |
| **LUMO (eV, 0 GPa)** | −5.49 | −5.36 | −5.34 |
| **HOMO (eV,** **0 GPa)** | −2.05 | −2.03 | −2.48 |
| **Band gap (eV, 0 GPa)** | 3.44 | 3.33 | 2.86 |
| **LUMO (eV**, **2 GPa)** | −5.51 | −5.51 | −5.46 |
| **HOMO (eV**, **2 GPa)** | −4.17 | −4.90 | −4.92 |
| **Band gap (eV**, **2 GPa)** | 1.34 | 0.61 | 0.54 |

# 4. References

1.	Y. Qin, J. Lu, C. Zhang, L. Xu, W.-Y. Wong, "Auxiliary Ligand‐Coordinated Nanoconfined Hydrophobic Microenvironments in a Nickel (II)‐Acetylide Framework for Enhanced CO_2_ Photoreduction", *Angewandte Chemie International Edition* 64 *(*2025): e202505883.

2.	M. Zhu, H. Su, F. Yang, Y. Qin, F. C.-H. Hui, R. Zhang, J. Geng, K. Wang, X. Fan, W.-Y. Wong, L. Xu, "Piezoelectrically Enhanced Charge Carriers Transfer in a Highly Conjugated Nickel (II)-Acetylide Framework for Photocatalytic CO_2_ Reduction", *Journal of the American Chemical Society* 147 *(*2025): 29192-29204.

3.	Y. Qin, Y. Wang, J. Lu, L. Xu, W. Y. Wong, "A Highly Conjugated Nickel(II)‐Acetylide Framework for Efficient Photocatalytic Carbon Dioxide Reduction", *Angewandte Chemie International Edition* 64 *(*2024): e202418269.

4.	L. Liu, M. Ruan, C. Wang, Z. Liu, "Optimization of the BiO_8_ Polar Group of BiVO_4_ by Cl--Embedded Modification to Manipulate Bulk-Surface Carrier Separation for Achieving Efficient Piezo-Pec Water Oxidation", *Applied Catalysis B: Environment and Energy* 354 *(*2024): 124117.

5.	S. Lin, C. S. Diercks, Y.-B. Zhang, N. Kornienko, E. M. Nichols, Y. Zhao, A. R. Paris, D. Kim, P. Yang, O. M. Yaghi, "Covalent Organic Frameworks Comprising Cobalt Porphyrins for Catalytic CO_2_ Reduction in Water", *Science* 349 *(*2015): 1208-1213.

6.	G. Kresse, J. Furthmüller, "Efficient Iterative Schemes for Ab Initio Total-Energy Calculations Using a Plane-Wave Basis Set", *Physical Review B* 54 *(*1996): 11169.

7.	X. Zhao, J. Chen, Z. Bi, S. Chen, L. Feng, X. Zhou, H. Zhang, Y. Zhou, T. Wågberg, G. Hu, "Electron Modulation and Morphology Engineering Jointly Accelerate Oxygen Reaction to Enhance Zn‐Air Battery Performance", *Advanced Science* 10 *(*2023): 2205889.

8.	D. J. Flannigan, K. S. Suslick, "Plasma Formation and Temperature Measurement During Single-Bubble Cavitation", *Nature* 434 *(*2005): 52-55.

9.	J. Tan, Y. Wang, Z. Wang, X. He, Y. Liu, B. Wang, M. I. Katsnelson, S. Yuan, "Large out-of-Plane Piezoelectricity of Oxygen Functionalized Mxenes for Ultrathin Piezoelectric Cantilevers and Diaphragms", *Nano Energy* 65 *(*2019): 104058.

10.	J. Xu, H. Che, C. Tang, B. Liu, Y. Ao, "Tandem Fields Facilitating Directional Carrier Migration in Van Der Waals Heterojunction for Efficient Overall Piezo‐Synthesis of H_2_O_2_", *Advanced Materials* 36 *(*2024): 2404539.

11.	Y.-Q. Li, Q.-W. He, D.-S. Tang, X. Shang, X.-C. Wang, "Intrinsically Asymmetric Atomic Character Regulates Piezoelectricity in Two-Dimensional Materials", *Frontiers of Physics* 19 *(*2024): 33201.

12.	Q. Yang, D. Wang, Z.-Y. Zeng, H.-Y. Geng, X.-R. Chen, "High-Performance Photocatalytic and Piezoelectric Properties of Two-Dimensional Transition Metal Oxyhalide ZrOX_2_ (X= Br, I) and Their Janus Structures", *Physical Review B* 109 *(*2024): 035411.

13.	H. Hallil, W. Cai, K. Zhang, P. Yu, S. Liu, R. Xu, C. Zhu, Q. Xiong, Z. Liu, Q. Zhang, "Strong Piezoelectricity in 3r‐Mos2 Flakes", *Advanced Electronic Materials* 8 *(*2022): 2101131.

14.	S. Yuan, Y. Zhang, M. Dai, Y. Chen, H. Yu, Z. Ma, W. F. Io, X. Luo, P. Hou, J. Hao, "A Giant Tunable Piezoelectric Performance in Two‐Dimensional In_2_Se_3_ Via Interface Engineering", *Advanced Electronic Materials* 10 *(*2024): 2300741.

15	P.-K. Yang, S.-A. Chou, C.-H. Hsu, R. J. Mathew, K.-H. Chiang, J.-Y. Yang, Y.-T. Chen, "Tin Disulfide Piezoelectric Nanogenerators for Biomechanical Energy Harvesting and Intelligent Human-Robot Interface Applications", *Nano Energy* 75 *(*2020): 104879.

16	H. Che, X. Wang, H. Yue, C. Chen, D. Xie, S. Yang, B. Liu, Y. Ao, "Precise Regulation of D‐Band Centers Inducing to High‐Efficiency Dual‐Channel Piezocatalytic H_2_O_2_ Production", *Advanced Functional Materials (*2025): e16979.

17	Q. Gu, X. Lu, C. Chen, X. Wang, F. Kang, Y. Y. Li, Q. Xu, J. Lu, Y. Han, W. Qin, "High‐Performance Piezoelectric Two‐Dimensional Covalent Organic Frameworks", *Angewandte Chemie International Edition* 63 *(*2024): e202409708.

18	H. Hu, X. Li, K. Zhang, G. Yan, W. Kong, A. Qin, Y. Ma, A. Li, K. Wang, H. Huang, "Dual Modification of Metal–Organic Frameworks for Exceptional High Piezo‐Photocatalytic Hydrogen Production", *Advanced Materials* 37 *(*2025): 2419023.
